# A Colloidal Quantum Dot Thermistor and Bolometer

**DOI:** 10.1002/adma.202519385

**Published:** 2026-05-04

**Authors:** Gaurav Kumar, Mariona Dalmases, Nima Taghipour, Rajesh Bera, Guy L. Whitworth, Goretti Torres Perez, Miguel Dosil, Gerasimos Konstantatos

**Affiliations:** ^1^ ICFO‐Insitut de Ciencies Fotoniques The Barcelona Institute of Science and Technology Barcelona Spain; ^2^ ICREA‐Institució Catalana de Recerca i Estudiats Avançats Barcelona Spain

**Keywords:** bolometer, colloidal quantum dot, metamaterial absorber, potential barrier, thermistor

## Abstract

Bolometric detection offers a compelling route to room‐temperature mid‐ and long‐wave infrared (MWIR/LWIR) photodetection by measuring temperature‐induced conductivity changes in a thermistor element thermally coupled to an absorber. However, conventional thermistor materials such as vanadium oxide (VO_x_) and amorphous silicon (a‐Si) exhibit moderate temperature coefficient of resistance (TCR) values (−2 to −3%/K). Higher TCRs have been achieved using SiGe/Si quantum wells (∼−5%/K), yet these require costly epitaxial growth and further improvements are hindered by lattice mismatch‐induced defects. Here, we report a novel thermistor platform based on colloidal quantum dots (CQDs) that circumvents these limitations by exploiting their lattice‐mismatch‐free nature. By tuning the size and surface chemistry of lead chalcogenide CQDs, we engineer the energetic potential landscape to modulate thermal activation energy, achieving TCR values of up to −9%/K. We further integrate this CQD thermistor with a plasmonic metamaterial absorber (PMA), enabling room‐temperature wavelength‐selective photodetection across the mid‐ to long‐wave infrared (MWIR/LWIR) spectrum. The bolometer detectors exhibited LWIR response with a time constant of ∼8 ms and room‐temperature detectivity approaching 10^6^ Jones at 9 µm, without using microelectromechanical systems (MEMS) technology.

## Graphical abstract

This work introduces colloidal quantum dot thermistors employing a potential barrier structure to tune the activation energy of transport and hence the temperature coefficient of resistance (TCR). Upon integration with plasmonic absorbers, the CQD‐based bolometer device enables room‐temperature wavelength‐selective photodetection across the mid‐ to long‐wave infrared (MWIR/LWIR) spectrum.

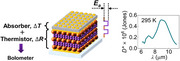

## 1 Introduction

Photon detection in the mid‐and long‐wave infrared (MWIR/LWIR) is of paramount importance for various applications related to health, safety, surveillance, process control monitoring, automotive safety, gas and environmental monitoring, and thermal imaging, just to name a few. Infrared photodetection has traditionally relied on costly epitaxial III–V [1, 2] and II–VI [3] semiconductors. These photodetectors also require cryogenic cooling and are therefore limited to niche metrology and military applications. There is an indispensable need for low‐cost, scalable, and CMOS‐compatible infrared detection technology to unleash its potential in volume markets. Colloidal quantum dot (CQD) technology has emerged to revolutionize the optoelectronic industry as a low‐cost CMOS‐compatible material platform. LEDs [4], photodetectors [5], image sensors [6, 7], and lasers [8] have been reported based on CQDs from the visible up to MWIR spectrum. While for SWIR applications, CQD photodetectors have demonstrated compelling performance at room temperature, longer wavelength detection still imposes the need for cryogenic cooling conditions similar to their single‐crystalline epitaxial counterparts. MWIR CQD photodetectors have been reported up to 6 µm, relying on cooling [9, 10, 11, 12, 13], and with room temperature operation up to 4 µm [14, 15]. Photodetectors with a response to longer wavelengths toward LWIR require cryogenic cooling with temperatures < 85 K [16, 17]. The need, therefore, for a low‐cost, scalable thermal sensor technology operating at room temperature still remains. We therefore took the view that in order to develop a practical MWIR‐LWIR and thermal sensing CQD technology, we needed to develop a new class of thermal sensor based on bolometer detection.

Bolometers are a class of thermal detectors comprising a thermistor element thermally coupled to an absorber, and photodetection is based on the measurement of conductivity change due to the temperature rise in the thermistor from the heat generated in the absorber. As such, bolometers can operate at room temperature. One of the key determining factors for the performance of a bolometer is its thermistor element and particularly the temperature coefficient of resistance (TCR) given by (1/*R*(*dR*/*dT*)), and noise. The implementation of thermistors to date has relied on the use of semiconductor materials with a disordered energetic potential landscape arising from spatial stoichiometric non‐uniformities or temperature‐induced phase transition, leading to high thermal activation energy (*E*_*a*_) of transport and thus high TCR. Representative materials include Vanadium oxide (VO_x_) [18], amorphous silicon (a‐Si) [19], as well as transition metal oxides [20, 21, 22, 23]. In addition to those, metal nanoparticle‐based thermistors have also been reported as thermocouples in which TCR is engineered by tuning the organic ligand length in view of the hopping nature of charge transport in those solids [24, 25]. Amongst them, VO_x_ and a‐Si, with a typical TCR of −2–3%/K have been currently used in commercial bolometer technology, and their performance has remained stagnant over the years. To reach even higher TCR values, SiGe/Si quantum well thermistors have also emerged as potential alternatives [26]. In such structures, a thin layer of Si_x_Ge_1‐x_ alloy is typically sandwiched between Si layers, and controlling Ge fraction allows for achieving high TCR values of ∼−5%/K [27]. However, such structures require epitaxy, leading to higher costs. Moreover, the lattice mismatch between Si and SiGe layers of more than 50% Ge content leads to defect dislocations preventing the realization of higher TCRs [27, 28, 29, 30]. Here, we overcome this barrier by exploiting the lattice‐matching‐free condition of CQDs in modulating the energetic potential landscape by tuning CQD size and surface chemistry in order to engineer the thermal activation energy of carrier transport and thereby the TCR. Hence, we demonstrate an IR bolometer device, where a CQD thermistor has been developed by vertically stacking differently‐sized CQDs to form a potential barrier structure that allows efficient modulation of *E_a_* and hence TCR, leading to TCR values as high as −9%/K. We then integrated our CQD thermistor with a plasmonic metamaterial absorber (PMA) structure to demonstrate room temperature photodetection tunable across the MWIR/LWIR range.

## 2 Results and Discussion

### 2.1 Quantum Dot Potential‐Barrier Thermistor (QDPBT)

**FIGURE 1:**
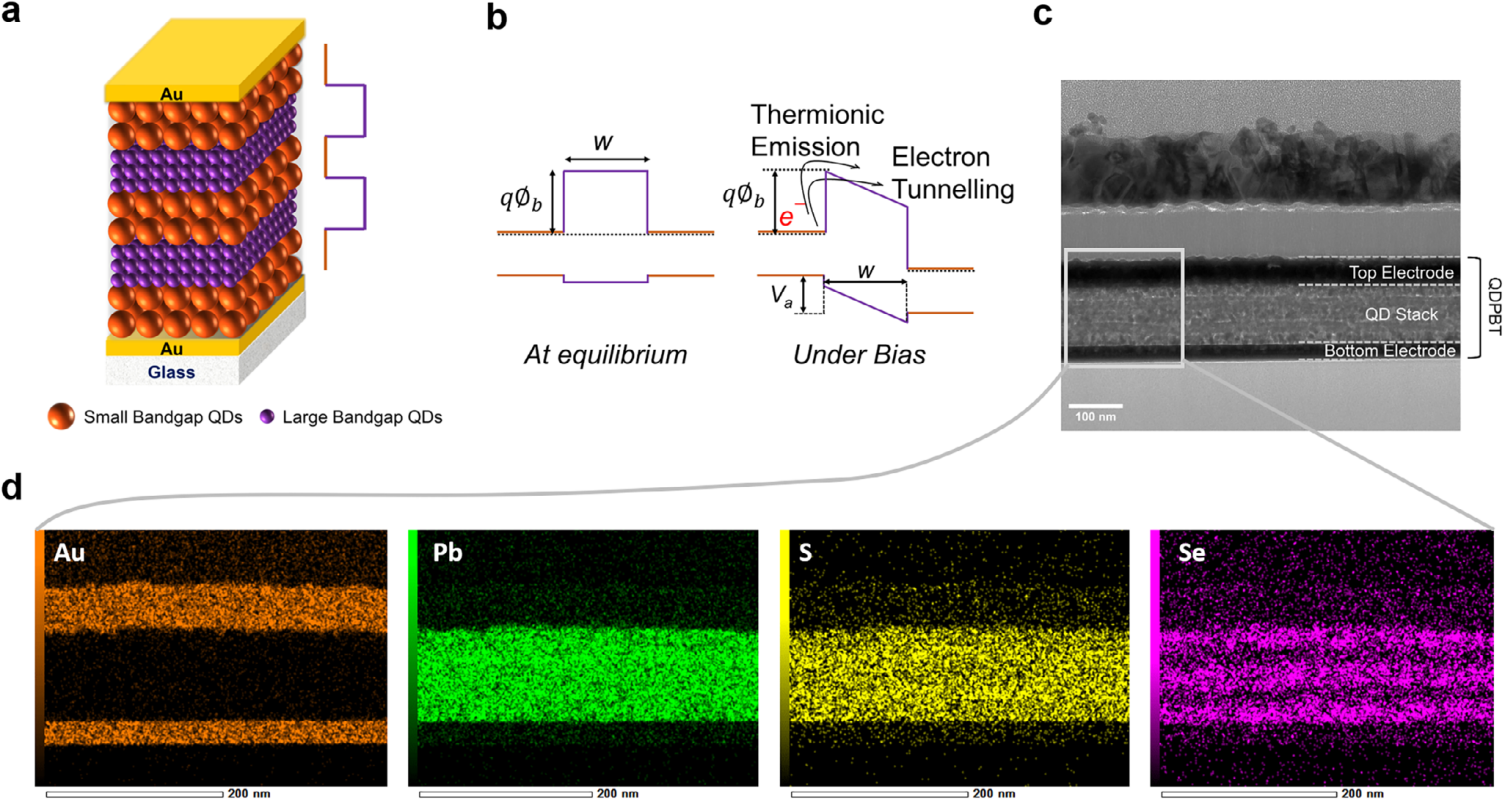
Design and working mechanism of the CQDs QDPBT. (a), Schematic of the QDPBT structure with Au electrodes on top and bottom to achieve symmetric behavior, alongwith a representative conduction band alignment to depict the scheme. (b), The charge carrier conduction mechanism of the PB‐type thermistor structures, without bias (at equilibrium) and with applied bias showing the interplay of two dominant mechanisms of carrier activation i.e. thermionic emission and thermionic field emission as a result of electron tunnelling through the narrowed PB. (c), High‐resolution transmission electron microscopy (HR‐TEM) image of the cross‐section of the QDPBT structure. The QDPBT layer is visibly made up of layers with large QDs and small QDs. The other visible layers in the image are the metal and dielectric spacer (discussed later). (d), Energy dispersive spectroscopy (EDS) based elemental mapping images of the QDPBT structure.

A QDPBT structure developed here, as shown in Figure 1a, addresses the above‐mentioned challenges, where the use of CQDs provides facile tuning of electronic properties, lattice‐mismatch‐independent growth, and therefore offers a high degree of freedom for tailored performance. Lead chalcogenide CQDs were chosen for this purpose because they remain the most studied material system for which surface chemistries have been developed to tune the doping [31] and band level positions of CQD films [30].

The QD‐PBT structure consists of alternate layers of larger‐sized PbS/PbSSe CQDs (∼7 nm) and smaller‐sized PbS CQDs (∼3 nm) (Figures S1 and S2 TEM and absorption). The larger‐sized CQDs with a smaller bandgap serve as the contact and high conductivity layers, whereas the smaller‐sized CQDs form the potential barriers (PB) for the charge carriers, owing to energy level modifications related to the size and the type of ligands of the QDs [32, 33] (UPS data, Figure S3). For the large CQD layers, we opted for the PbS/PbSSe core‐alloyed shell structure in view of its higher doping compared to PbS CQDs [34]. A representative conduction band alignment alongside the QDPBT schematic displays this scheme, where the energy levels are aligned in a way to have an offset in the conduction bands of the two adjacent layers, resulting in a PB for electrons to overcome, and consequently an electron‐limited charge conduction. The QDPBT structure was prepared by simple layer‐by‐layer deposition of CQDs dispersed in toluene as solvent using spin coating technique, in contrast to epitaxial materials with energy‐consuming methods, followed by conventional ligand exchange and alumina coating processes (see methods). The high‐resolution transmission electron microscopy (HR‐TEM) image and the energy dispersive spectroscopy (EDS) based elemental analysis shown in Figure 1c,d shows the arrangement of CQDs layers with clearly defined boundaries. The pixel size in this study is 30 µm × 30 µm, defined by the top and bottom overlapping electrodes.

For QDPBT, the charge carriers confined by the PB take part in thermal activation, which can be visualized by the schematic shown in Figure 1b describing the approximate charge transport mechanism and the band alignment based on the provided UPS data (Figure S3). Assuming a small applied bias *V_a_*, only a few electrons can attain enough energy to cross the barrier by overcoming the PB height *q*∅_𝑏_ because of thermal activation, thus defining the *E_a_*. As *V_a_* is increased, the PB tilts and the electrons rising in energy find the PB width varying, giving rise to tunnelling of electrons through the reduced barrier width *w* at the top of the barrier. Akio Furukawa presented the theoretical calculation of such a PB structure and established the dependence of TCR on various parameters: barrier height *q*∅_𝑏_, barrier width *w*, and the bias applied *V_a_* [35]. Several experimental demonstrations involving similar structures have also shown similar dependence of the TCR [36, 37].

**FIGURE 2:**
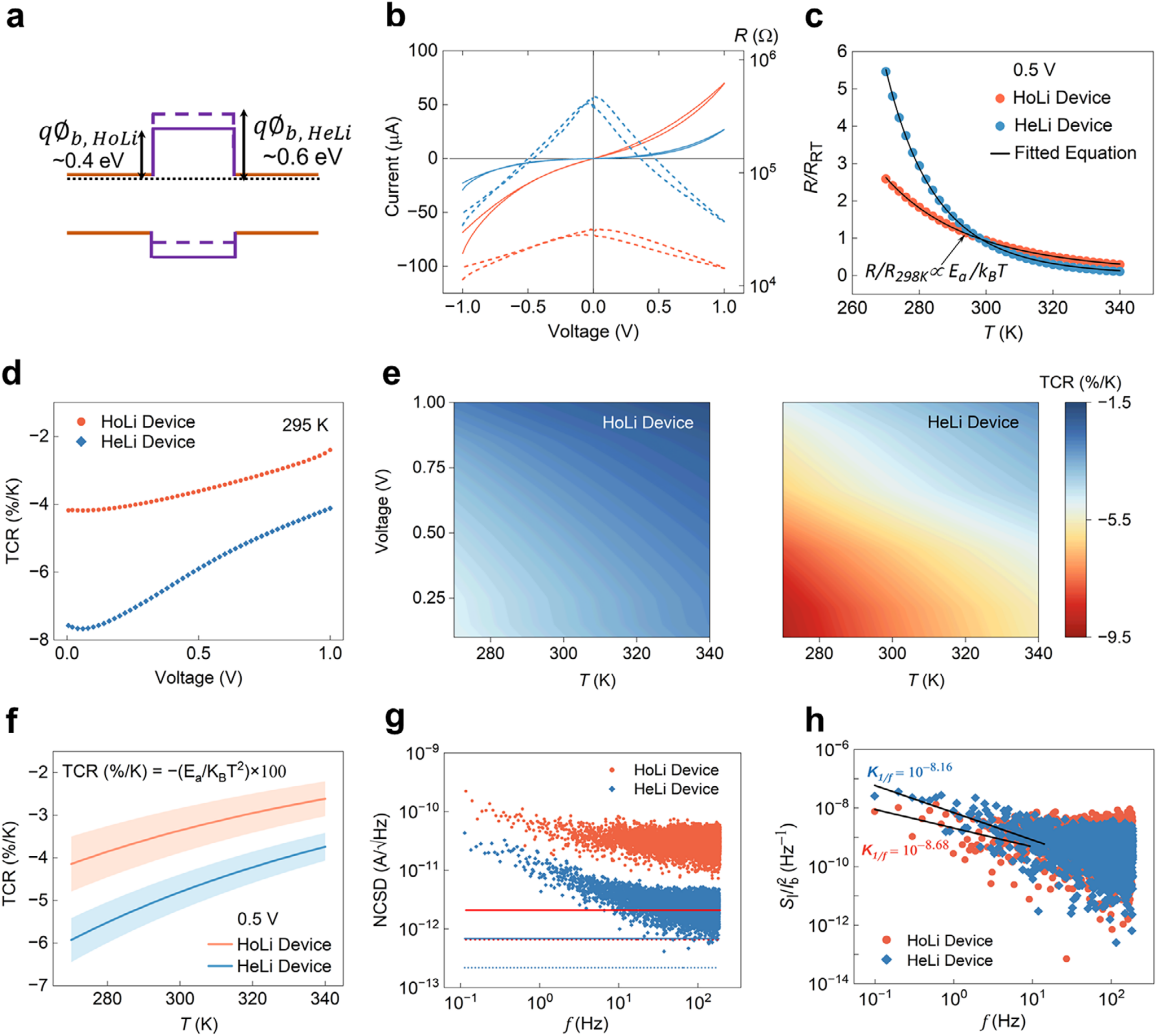
Temperature‐dependent electrical characterization results of the QDPBT structure. (a), Schematic band alignment illustrating the approximate relative positions of the energy levels of the large and smaller CQDs with HoLi and HeLi configurations as per the UPS measurements, at equilibrium. (b), Room‐temperature *I*–*V* characteristics of the HoLi (Orange) and HeLi (Blue) devices along with corresponding resistance are plotted here. The *I*–*V* curve signifies the symmetry of the device and resembles a PB‐type structure. (c), The variation of QDPBT resistance with temperature is plotted at a given bias of 0.5 V, for both HeLi and HoLi configurations. The experimental data is fitted with the Arrhenius equation to extract the E_a_. (d), TCR vs. *V* plot for the devices shows the variation of QDPBT's TCR at 295 K. TCR was extracted from the values of *E*_*a*_ by fitting the *R* vs. *T* plot at every voltage value. (e), Variation of TCR with the temperature and the applied bias for HoLi and HeLi configurations. (f), The statistical data of the QDPBT with HoLi and HeLi configurations, with the shaded portion representing the standard deviation and the solid line the mean. (g), The noise current spectral density of the devices, at an applied bias of 0.5 V, room temperature, and under vacuum conditions. (h), Current normalized power spectral density for the devices showing the K_1/f_ parameter.

Figure 2 presents temperature‐dependent electrical measurements for CQDs QDPBTs composed of large PbS/PbSSe core‐alloyed shell CQDs (exciton peak ∼1.95 µm) and smaller PbS core CQDs (exciton peak ∼0.85 µm). Large CQDs were treated with 1‐ethyl‐3‐methylimidazolium iodide (EMII) ligands for heavy doping, while smaller CQDs underwent ligand exchange based on EMII (in a device called homo‐ligand—HoLi) or 3‐mercaptopropionic acid (MPA) (in a device called hetero‐ligand—HeLi). The choice of EMII for the larger CQDs is to achieve heavy doping for larger CQDs [38], while the choice of MPA for HeLi configuration for smaller CQDs was made to achieve a higher conduction band offset between the two layers [32]. As shown in Figure 2a, the HeLi configuration yields a higher potential barrier compared to HoLi (band structure based on UPS data provided in Figure S3), resulting in increased *E_a_* and TCR. A small PB for the hole transport is evident also; however, its effect on TCR is predicted to be minimal due to a significantly large PB for electron transport. Figure 2b shows the room‐temperature bi‐directional *I*–*V* curve of a double PB (DPB) QDPBT device for both configurations, characterized by a symmetric behavior and non‐linear bias dependence due to the presence of PB layers in agreement with earlier studies [39, 40]. Notably, the DPB HeLi device's *I*–*V* curve exhibits a much higher resistance at a given voltage, in view of the larger PB height in the HeLi configuration compared to HoLi devices. Figure 2c shows the variation of resistance (*R*) as a function of temperature (*T*) for both configurations at a voltage of 0.5 V, evaluated from temperature‐dependent *I*–*V* curves (Figure S4). The QDPBT devices were tested for repeated temperature ramping and cooling cycles and were observed to be stable after the first ramping up cycle (Figure S5). The resistance of the HeLi device exhibits a stronger temperature dependence as compared to the HoLi device, which can be attributed to an increased number of confined charge carriers by the PB. *R* vs. *T* plot is typically used to identify the process by which charge carriers conduct through the CQDs film [41, 42]. Such investigations have shown the dominance of nearest‐neighbour hopping (NNH) type transport for a temperature range consistent with our studied temperature range, suggesting an Arrhenius‐type behavior of charge conductance (or resistance). This Arrhenius‐type behavior can be represented by *R*  = *R*_0_ *exp*(*E_a_*/*k_B_T*), where *R_0_* is the pre‐exponential factor, and *E_a_* is the activation energy of transport. Following this, fitting the *R* vs. *T* experimental data with the Arrhenius equation allowed us to extract the activation energy *E_a_* for the transport of charge carries at a particular voltage, as shown in Figure 2c. To confirm the charge transport mechanism, we have also attempted to fit our *R* vs. *T* data with variable‐range hopping (VRH), and found that the NNH type fit provided the best results, in agreement with the reported studies (Note S1). The value of *E_a_* thus estimated using NNH was found to be ∼0.27 and ∼0.42 eV for HoLi and HeLi configurations, respectively. The QDPBT devices in single PB (SPB) configuration with homogenous ligands as in HoLi were also studied to quantify the effect of the PB layer width and the number of PB layers on thermistor performance (Figures S8 and S9). It was observed that DPB configuration exhibited better performance in terms of TCR in comparison to SPB. It should be noted that the extracted *E_a_* value represents an effective *E_a_* faced by the charge carriers, which originates from various contributions, such as from the trap states, inter‐QD hopping barrier, and the inter‐QD PB height resulting from energy band offsets, which are being manipulated here. Another degree of freedom available with CQDs is the change in size of PB layer QDs, which is expected to influence the PB height due to a change in bandgap. To study this, DPB HeLi devices with varying size of PB layer QDs were also measured, where the *E_a_* was found to increase with decreasing size of PB layer QDs (Figure S10). To quantify and corroborate the role of QDPBT structure on the activation energy, we also studied films comprising the CQDs employed in our QDPBT structures individually (Figure S11). A higher value of *E_a_* for the QDPBT and its enhancement for HeLi configuration thus corroborates the presence of a much higher PB height resulting from the shift in the energy levels of the MPA‐treated smaller CQDs.

From the relation of *E_a_* with TCR, i.e., *TCR* (*K* ^−1^)  =   − *E_a_*/*k_B_T* ^2^, values of TCR were calculated at 295 K for every voltage, as shown in Figure 2d. It is evident from the plot that the HeLi configuration enhances the thermistor behavior of the QDPBT structure. For both configurations, TCR exhibits a voltage dependence and is found to be higher at lower voltages and lower at higher voltages, in accordance with the theoretical studies of Furukawa et al. [35]. Additionally, TCR was found to depend on the operating temperature as presented in the colour map plot in Figure 2e, for HoLi and HeLi configurations. The TCR maps in Figure 2e are calculated by fitting *R* vs. *T* data with the Arrhenius equation, at each applied voltage, to get *E_a_* vs. *V* data, which was used to plot the colour maps of TCR. The TCR is higher at lower temperatures and lower applied bias. The TCR ranges between − 1.8 and   − 5.0 %/K for the HoLi device whereas it ranges between − 3.1 and − 9.1 %/K for the HeLi configuration, for the studied temperature range of 270 – 340 K, and the voltage bias of 0.1 – 1.0 V. State‐of‐the‐art (SOA) Si/SiGe QW thermistors [27], have demonstrated a TCR value of − 5.5 %/K with a pixel resistance of 4.4 × 10^6^ Ω and a pixel size of 25 µm × 25 µm at an applied bias of 0.33 V. The corresponding values for our QDPBT for the same applied bias are about −6.7 %/K with a pixel resistance of ∼9 × 10^6^ Ω for a pixel size of 30 µm × 30 µm. This comparison highlights the compelling performance of our solution processed thermistors over their epitaxial counterparts. Several devices were fabricated and characterized for both HoLi and HeLi configurations and the statistical TCR vs. *V* data is shown in Figure 2f.

Another critical figure of merit for assessing the sensitivity of a sensor is its noise. The noise current spectral density (NCSD) for the QDPBT is shown in Figure 2g. It is evident that for lower frequencies (< ∼10 Hz), the NCSD spectrum is dominated by the flicker or *1/f* noise, and at higher frequencies becomes equal to a white noise level. The observation of the *1/f* noise dominance at low frequency is consistent with the results of several studies, in which the fluctuations in the mobility, the granular nature of the nanocrystals, and the presence of traps and defects have been identified as the primary source of the noise in nanocrystals [43, 44]. The Johnson or the thermal noise (dashed lines) and the shot noise levels (solid lines) are also marked in the plot according to: $NCSD^{Thermal}=\sqrt{S_{I}^{Thermal}}=\sqrt{4k_{B}T/R}$ , and $NCSD^{Shot}=\sqrt{S_{I}^{Shot}}=\sqrt{2eI_{d}}$ , respectively, where *S_I_* is the noise power spectral density (NPSD), *e* is the electronic charge, *I_d_* is the dark current, and *R* is the resistance of the device. Typically, the *1/f* noise is characterized by $S_{I}^{1/f}=K_{1/f}\;I^{\beta }/f^{\gamma }$, where *K_1/f_* is the *1/f*‐parameter, that can be used to compare noise characteristics across the different configurations. In Figure 2h, we plot the current‐normalized NPSD, assuming a homogeneous nature of QDPBT, i.e. β∼2 as a function of *f*. The *K_1/f_* has been estimated for both HoLi and HeLi configurations based on the log‐log slope, to be ∼2.1 × 10^−9^ and ∼6.9 × 10^−9^, respectively. We have also provided the device‐to‐device statistics for five devices of HoLi and HeLi configurations each, and have calculated the *K_1/f_* factor and demonstrated the statistics in the form of a box plot (Figure S12). The HeLi configuration was found to have a slightly higher 1/*f‐*noise, which could be attributed to morphological parameters, such as the interface quality, which is believed to be higher in the HoLi configuration due to the homogeneity of the solvents used. In addition, Figure S10c shows that the noise further increases for DPB configuration with the reduction in the size of the PB layer QDs. Furthermore, we have shown the bias dependence of noise for HoLi and HeLi configuration devices (Figure S13), and have compared the noise at 1 Hz for both configurations (Table S2).

### 2.2 Quantum Dot Potential‐Barrier (QDPBT) Bolometer

**FIGURE 3:**
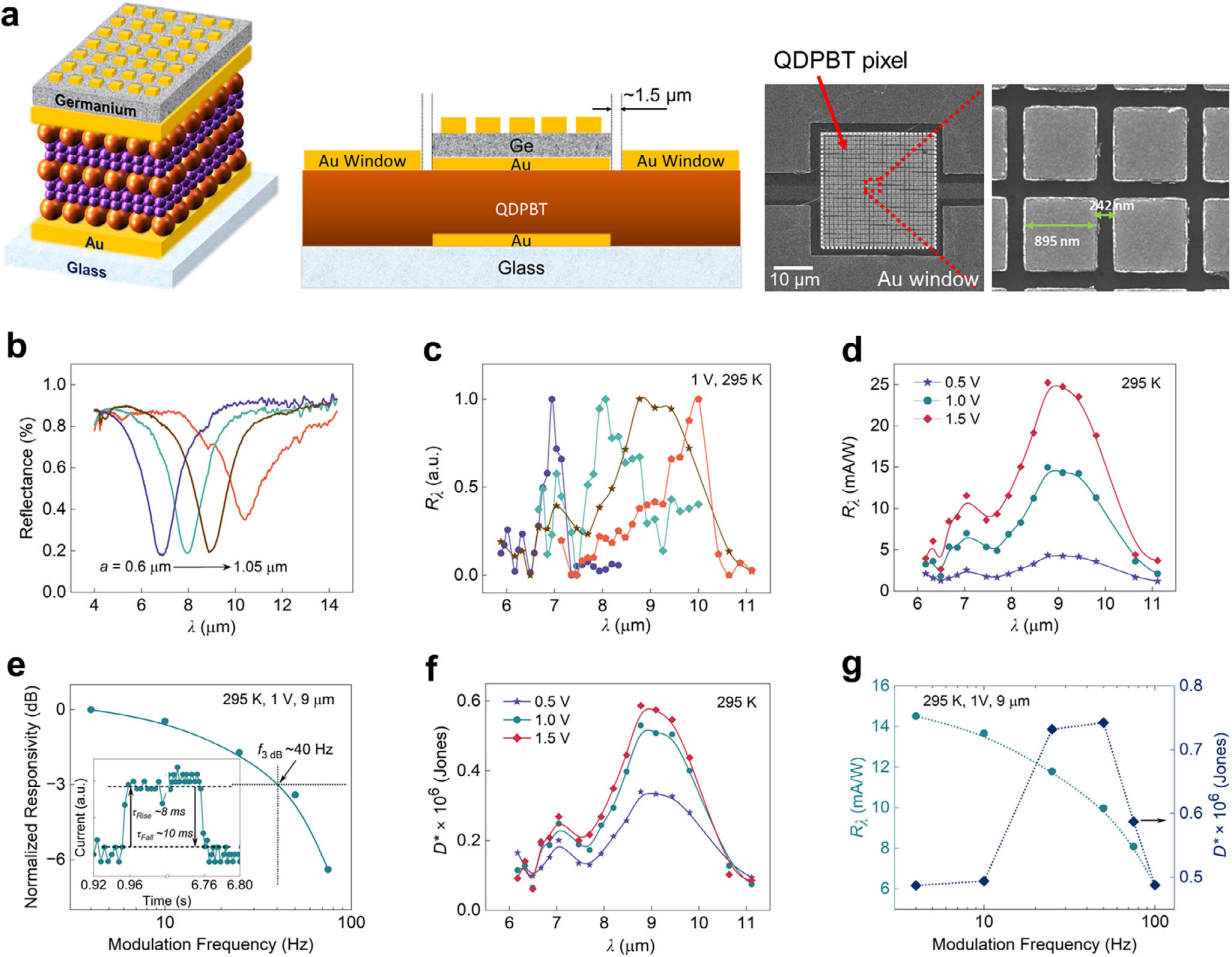
The CQD bolometer device. (a), Schematic of the bolometer device with a PMA structure fabricated on top of the QDPBT structure. The SEM image shows the QDPBT pixel with the PMA pattern and the Au window surrounding it, including a magnified view of the metamaterial pattern. (b), Demonstration of the variation of the resonant peak of the PMA with changing size of the metamaterial pattern. The figure shows the measured FTIR spectra of the fabricated PMAs with size a, varying from 0.6 to 1.05 µm, keeping spacing constant as 0.25 µm, dielectric thickness of ∼80 nm, and top metamaterial pattern thickness of ∼130 nm. (c), Demonstration of multiwavelength sensing by the CQD bolometer device. (d), Responsivity of the bolometer device as a function of the wavelength of the IR light, respectively, for a resonant peak at ∼9 µm wavelength, at room temperature and vacuum conditions. (e), The frequency response of the bolometer device with the time response shown in the inset. (f), The *D* ^*^ of the device at several applied voltage biases. (g), The dependence of the device's responsivity and detectivity on the light modulation frequency.

To enable long‐wave IR sensing, a plasmonic metamaterial absorber (PMA) structure was integrated with the QDPBT, as depicted in Figure 3a. The complete structure of the bolometer device is visible in the HR‐TEM image shown in Figure 1c and also in the Figure S14, which shows the elemental analysis of the different layers. An opaque Au window surrounding the QDPBT can also be seen in the SEM image (in Figure 3a) of the pixel, which helps in minimizing the contribution of area other than the QDPBT pixel area in the thermal response due to unwanted IR absorption (Figure S15). The PMA structure consists of a ground metal plate, a dielectric spacer Ge (Figures S16 and S17), and a metamaterial pattern on top, as shown in Figure S18a. Such PMAs have been utilized and proposed in the past in several studies for their suitability to IR sensing [45, 46]. The sensing mechanism by the PMA is explained in Note S3. The resonant peak of such an absorber can easily be tuned by varying parameters such as the size *a*, spacing *s*, thickness of dielectric *d*, etc., as demonstrated in Figure 3b, where we have varied the size *a* (Figure S18d). Responsivity is an important figure of merit for optical sensors, which can be given by, *R*_λ_ = Δ*I*/*P_in_*  where Δ*I* is the measured change in the device current upon illumination (in Amperes), and *P_in_* is the input optical power (in Watts). By integrating those PMAs on our thermistors, we demonstrate the possibility of wavelength‐selective detection across the MWIR/LWIR, as shown by the normalized responsivity of various pixels with PMA resonances tuned at different wavelengths in Figure 3c. We then attempted to quantify the responsivity and sensitivity of our bolometers. To ensure operational stability, the devices were subjected to bias for ∼3–4 min to minimize the current drift and provide a stable response (Figure S20). The IR source, i.e. QCL's beam spot measurement and the estimation of the power transmitted through the Au window can be found in the Note S4 and Figures S21 and S22. The current signal of the device with PMA having a peak at ∼9 µm wavelength under optical illumination by QCL is shown in the Figure S23. The current spectra shown is for a HoLi configuration with a similar TCR and resistance values as described earlier (Figure S24). For the bolometer demonstration we opted for the HoLi configuration in view of its lower noise and better film quality increasing the yield of the fabrication process of the PMA atop the thermistor films. Figure 3d illustrates the *R*_λ_ of the device as a function of the wavelength, *λ* of IR light. The optical power dependent photocurrent and *R*_λ_ for the device shown in Figure S25, exhibits linear response akin to a bolometer detector. The device frequency response was also studied, as shown in Figure 3e, where the inset shows the time response of the device to switching optical excitation. The 3 dB bandwidth of the device, *f*_3*dB*_ was found to be around 40 Hz, roughly correlating with time response τ_*rise*_ ∼8 ms through the relation $f_{3dB}∼0.35/\tau$. Despite the fact that our bolometer is in thermal contact with the substrate, thus suffering from large thermal capacitance, its time response is comparable to commercial SOA bolometers [47, 48, 49], albeit those are on MEMS suspended membrane structures to ensure thermal isolation and therefore minimize thermal capacitance. The device is found to be reasonably stable for longer periods of time (∼4 months) when stored under glove box or desiccator (Figure S26).

We last sought to assess the detectivity of our bolometers, $D^{*}=R_{\lambda }\sqrt{A_{d}}/I_{n}/B$ , where *R*_λ_ is the responsivity in AW^−1^, *A_d_* is the device area (in cm^2^), and *I_n_*/*B* is the noise current spectral density (NCSD) (in *A/* $\sqrt{Hz}$) (Figure S27), plotted in Figure 3f. The value of D* was found to be in the range of 0.5 × 10^6^ Jones for our device, under 1 V operation. The dependence of detectivity on the frequency of operation shown in Figure 3g shows that the peak detectivity of about 0.75 × 10^6^ Jones is achieved around *f*_3*dB*_. We further calculated the noise equivalent power (NEP) of our device around the *f*_3*dB*_, from its relation with D^*^, i.e., $NEP=\sqrt{A_{d}}/D^{*}$ $\left(W/\sqrt{Hz}\right)$. The NEP was estimated to be ∼4 $nW/\sqrt{Hz}$, at 1 V and *f*_3*dB*_.

The bolometer in this work is directly atop the substrate, i.e. not thermally isolated in a suspended structure. This has important implications to its performance due to the thermal coupling losses. Considering the absorbed optical power by the PMA, TCR, and the observed current change Δ*I*, we have estimated the temperature rise, Δ*T*, and the thermal conductance, *G_th_* to be around 230 mK and 5 × 10^−5^ W K^−1^, respectively (Note S5). The estimated value of *G_th_* is characteristic value for such substrate‐coupled configuration and is three orders of magnitude higher than the *G_th_* of MEMS bolometers on suspended structures [50, 51, 52, 53]. Furthermore, *R*_λ_ is related to the input optical power *P_in_*, TCR, and the Δ*T* by: *R*_λ_ = Δ*I*/*P_in_*  = (α × *I_d_* × Δ*T*/*P_in_*) , and was estimated to be ∼14.5 mA/W at 1 V bias. The low D^*^ and high *G_th_* values are typical for substrate‐coupled bolometer devices [54, 55, 56]. In addition, the SOA bolometers utilize suspended structures and demonstrate D^*^ values of 10^9^ Jones typically [57, 58, 59]. We expect, therefore, orders of magnitude improvement in the detectivity of our bolometers upon utilizing thermal isolation techniques commonly used in commercial bolometers.

## 3 Conclusion

In this study, we demonstrate a novel room‐temperature infrared (IR) bolometer based on a quantum dot potential‐barrier thermistor (QDPBT) structure utilizing colloidal quantum dots (CQDs). By vertically stacking differently sized lead chalcogenide CQDs to create engineered potential barriers, we achieve precise modulation of activation energy (*E_a_*) and, consequently, the temperature coefficient of resistance (TCR). The QDPBT devices exhibit tunable TCR values ranging from −3.1 to −9.1%/K, rivalling state‐of‐the‐art Si/SiGe quantum well thermistors, but with the added advantages of solution‐processability, low cost, and freedom from lattice mismatch constraints. The fabricated thermistors have been demonstrated to be readily integrable with nanophotonic PMAs, enabling room‐temperature LWIR with a detectivity approaching 10^6^ Jones and ∼8 ms time response, in the absence of advanced pixel isolation strategies using MEMS technology. The work presented here lays the foundation of a new type of material platform to advance uncooled IR sensing and broadens the application scope of CQD technology.

## 4 Methods

### 4.1 Colloidal Quantum Dots Synthesis

#### 4.1.1 PbS QDs With 850 nm excitonic peak

PbS QDs were synthesized under an inert atmosphere by hot injection method. 446 mg lead (II) oxide (PbO), 18 mL 1‐octadecene (ODE) and 1.6 mL oleic acid (OA) were degassed for 1 h under vacuum at 100 °C. Once under argon, the temperature was set at 80°C, and 210 µL hexamethyldisilathiane (HMS) in 5 mL ODE was quickly injected. After the injection, the reaction was cooled down naturally to room temperature. The PbS QDs were purified by the addition of a mixture of acetone/ethanol, redispersing with anhydrous toluene; this cleaning process was repeated three times. Finally, the concentration was adjusted to 30 mg/mL and the sample was stored at low temperature to avoid Ostwald ripening.

#### 4.1.2 PbS@PbSe Core–Shell QDs

PbS core (excitonic peak of 1800 nm) was synthesized under an inert atmosphere by the hot injection method. Briefly, 1.032 g PbO, 150 mL ODE, and 9.8 mL OA were heated at 100°C under vacuum for 1 h to form lead oleate. Under argon, the temperature was set at 115°C, and a solution of 135 µL HMS dissolved in 3 mL ODE was quickly injected. After 6 min of reaction, a second solution of 352 µL HMS in 9 mL ODE was dropwise injected for 22 min. Afterward, the solution was allowed to cool down naturally. Once at room temperature, it was washed three times with a mixture of acetone/ethanol, redispersing in toluene. The final sample was redispersed in degassed ODE, and the concentration was adjusted to 70 mg/mL. This solution was stored at low temperature and under nitrogen. To grow a PbSe shell on these PbS core QDs, 660 mg PbO, 20 mL ODE, and 4.1 mL OA were heated to 100°C under vacuum for 1 h, to form the lead precursor. After the solution was changed to an argon atmosphere, the temperature was set to 150°C, and 1.4 mL of PbS‐ODE solution was injected. When the temperature was recovered, the selenium precursor was injected (289 mg Se powder dissolved in 2.9 mL tributylphosphine), and the temperature was kept constant at 150°C for 35 min. After this time, the reaction was quenched with a water bath. The QDs were washed three times with ethanol, redispersing in toluene. Finally, the concentration was adjusted to 30 mg/mL.

### 4.2 Device Fabrication

To make the QDPBT structure, clean glass substrates were used after ultrasonication‐assisted cleaning in acetone and IPA for 5 min each, followed by oxygen plasma cleaning. The bottom electrodes were patterned by photolithography using Heidelberg's MLA 150. The photoresist ECI 3007 was spin‐coated on the clean glass substrates at 4000 rpm, followed by baking at 100°C for 90 s before exposure. The exposed photoresist was developed using AZ MIF 726 developer solution for 30 s. To form the bottom electrodes, Ti (3 nm)/Au (27 nm) was evaporated using an e‐beam/thermal source and subsequently lifted off in acetone to leave patterned substrates. After that, the QD layers constituting the QDPBT structure were formed in a layer‐by‐layer manner using spin coating. First, the QD solution with large PbS/PbSSe core‐alloyed shells was dropped on the substrate and spun for 30 s at 2500 rpm, followed by ligand exchange with EMII for 30 s, before rinsing with methanol during spinning to remove excess and unbound ligands, resulting in a ∼25 nm film. To form PB layer, QDs with 850 nm exciton peak were spin‐coated at 2000 rpm and ligand‐exchanged with EMII for HoLi and 0.1% MPA for HeLi configuration, resulting in a film thickness of ∼25–30 nm. After this, one more layer of larger core–shell QDs to make the targeted QDPBT stack for a single potential barrier (SPB) configuration. Subsequently, one more layer of PB layer QDs followed by larger core‐alloyed shell QDs was repeated for double potential barrier (DPB) configuration (total of 5 layers). Atomic layer deposition (ALD) of alumina (Al_2_O_3_) was then performed on the samples at 80°C to infill the QD layers and stabilize the electronic properties [38]. A GEMstar XT thermal ALD system from ARRADIANCE, equipped with trimethylaluminium (TMA), purchased from STREM Chemicals Inc., and Millipore DI H_2_O were used as the precursors for this purpose. Photolithography was again utilized to form the top electrodes by integrating the top alumina etch in the photoresist development step. Top electrodes, along with the reflection window, were made with Au (∼60 nm) in a single step, followed by lift‐off. To fabricate the absorber structure, device pixel‐sized windows were aligned and patterned in the photoresist using photolithography, followed by e‐beam evaporation of germanium (Ge) (∼80 nm) and the lift‐off method. The samples were then coated twice with 950k PMMA from EM Resist Ltd. by spin coating at 4000 rpm to form an approximately 750 nm thick PMMA film. The samples were then baked at 135°C for 60 s before being coated with a conductive polymer, Espacer 300Z from Resonac, to avoid charging issues. To make the PMMA pattern, FEI Inspect F50 e‐beam lithography was utilized to expose the pattern on the PMMA. The exposed pattern was developed using a 1:2 solution of DI water: IPA for 50 s. This was followed by depositing Ti (10 nm)/Au (120 nm) and subsequent lift‐off to form the complete bolometer device. The completed device includes seven pixels on a square glass substrate of ∼12 mm in size. The pixels are separated by ∼1 mm distance from one another, such that the distance between individual pixels is >> size of the pixel, so crosstalk is highly unlikely between the neighbouring PMA resonators. Moreover, as almost all the area around the pixels is shadowed by the Au reflection window, it does not allow the light to heat up the surrounding area.

### 4.3 Microscopy Characterization

For the TEM characterization of the QDs, a diluted solution was dropped on ultrathin carbon grids, followed by immersion in methanol for higher resolution. The TEM images were taken by using a JEOL 2100 microscope at the Scientific and Technological Centres of the University of Barcelona. The SEM images were taken using FEI Inspect F‐50 system. For the cross‐section HR‐TEM measurements, a lamella of 12 by 5 µm was prepared using FESEM‐FIB SCIOS and subsequently analyzed by FETEM JEOL F200 system for the cross‐section characterization.

### 4.4 Electrical Characterization

For electrical characterizations, the QDPBT device was placed in a cryostat and under dark conditions throughout the measurement. A B1500A semiconductor analyser unit was used to perform the source and measure operations. To study the temperature‐dependent behavior of the devices, current–voltage (*I*–*V*) characteristics were recorded in the temperature range of 270–340 K, with the help of an automatic test bench created on B1500A to control a Lakeshore 336 temperature controller. An ample relaxation time of about 12 min was provided in between each temperature setpoint to ensure a stable response of the device. The *I*–*V* data was then post‐processed with MATLAB to extract different parameters, such as *E_a_*. For the noise measurements, the device was placed in the shield chamber under vacuum, while the vacuum pumps were switched off and physical movements were minimized during the measurement. The dark current vs. time was scanned for a certain period for the device using a B1500A semiconductor analyzer, with a sampling interval of 2 ms, ADC integration factor and mode set to 1 and auto, respectively. For the concerned current ranges, these settings lead to a minimum integration time of 80 µs as per the instrument's user manual, with the final bandwidth determined by the sampling interval. To evaluate noise current density, Fast Fourier Transform (FFT) of the dark current scans was taken using a Hanning window and power correction, to ensure minimal spectral leakage due to the FFT process.

### 4.5 Optical and Spectroscopic Characterization

To ascertain the optical properties of the QDs, a Thermo Fisher Scientific‐made Nicolet iS50 FTIR was utilized in the ambient conditions. To measure the spectra of the CQD solution, a quartz cuvette was used, and the baseline was created with Toluene. A Bruker‐made verTera, along with Hyperion II, was utilized to qualify the reflection spectra of the PMAs. The aperture size during the measurement was kept close to the pixel size, and an objective of 36x was utilized. For reflection measurements, a standard Au sample from Bruker was used to take the baseline. The refractive index (*n*(λ)) and extinction coefficient (*k*(λ)) of Ge‐film were determined using variable‐angle spectroscopic ellipsometry, which is a sensitive and non‐destructive technique performed at room temperature (IR‐VASE, Mark II, J. A. Woollam Co.) (Note S2). The angle of incidence ranged from 55° to 75°, with a spectral resolution of 8 cm^−1^. The experimental data were subsequently fitted using a combination of Cody‐Lorentz and Gaussian oscillators to accurately fit the optical spectra of the films to extract *n‐k* values using Complete EASE software. The ultraviolet photoelectron spectroscopy (UPS) was performed with a SPECS PHOIBOS 150 hemispherical analyser under ultrahigh‐vacuum conditions (10^−10^ mbar) at the Institut Català de Nanociència i Nanotecnologia (ICN2) using He‐1 source (21.2 eV).

### 4.6 Lumerical FDTD Simulation

Optical simulations of the PMA structure were performed using the commercial software Ansys Lumerical FDTD. Due to the thick bottom Au layer, transmission through the PMA is negligible, so the underlying structure does not affect the results. The simulation model consists of a thin Au layer (50 nm) on top of a semi‐infinite Si substrate (z ≤ 0), with the Ge layer extending in the +z direction to the desired thickness, using measured *n‐k* values for material properties. A 10 nm Ti /120 nm Au block, representing the metamaterial pattern, is placed atop the Ge spacer. Field distributions are recorded using frequency‐domain fields and power monitors. Perfectly matched layer (PML) boundary conditions are applied along the ±z directions, while periodic boundary conditions are set along the ±x and ±y directions. A plane wave is injected in the –z direction.

### 4.7 Bolometer Device Characterization

The device was placed in a vacuum chamber with shielded metallic walls to avoid electromagnetic interference. Block Engineering's Laser Tune QCL is used as the IR source, which illuminated the sample through a 3 mm thick ZnSe window. The power of the laser was measured using a thermal power sensor S401C from ThorLabs connected to a ThorLabs power meter PM100D. The sensor was placed at the same spot in the probe station chamber as the actual sample to ensure reliable measurement of the laser power. To measure the spot size of the QCL, a pyroelectric camera from mks‐Ophir photonics (Pyrocam III HR Laser Beam Profiler) was utilized. The camera was placed at an approximately similar distance as the sample position during the measurements. The QCL beam was made to fall on a ThorLabs‐made 90° off‐axis parabolic Au mirror with a reflected focal length of about 10.1 cm and then illuminate the camera, in a manner like the bolometer device measurement setup. The device was biased using an SRS‐made low noise current pre‐amplifier SR570, which also collects the signal from the device simultaneously to deliver the signal to a Zurich Instruments MF‐Lock‐in amplifier. The reference signal to the lock‐in amplifier was provided by a Newport 3502 Optical Chopper supply, which controls the mechanical chopper with a duty cycle of 50%. The time constant of the low‐pass filter of the lock‐in amplifier was kept between 150–250 ms, to ensure a stable signal. The induced change in the device current Δ*I* was then extracted from the measured voltage signal in the lock‐in amplifier by:

$$\Delta I=\frac{2\pi \sqrt{2}V_{L}}{4}\;S$$

where, *V_L_* is the voltage signal measured by lock‐in amplifier in *volts*, *S* is the sensitivity of the pre‐amplifier in A/V, the factor *2* and $\sqrt{2}$ originates from the peak‐to‐peak magnitude and lock‐in amplifier rms amplitude, and π/4 comes from the fundamental sine‐wave Fourier component of the square wave due to the optical chopper [60].

The device was biased at a constant voltage and the QCL beam was chopped continuously to record the time and frequency response by B1500 Semiconductor analyser.

For the noise measurements of the device, dark current was recorded for a given time with a sampling frequency of 500 Hz. FFT analysis with Hanning window of the dark current using MATLAB provided the NCSD of the device.

## Author Contributions

G.K. conceived the idea, supervised and directed the study. G. K. and Ga.K. designed the experiments and co‐wrote the manuscript, with feedback from the co‐authors. Ga.K. developed the device layout and geometry, micro/nano‐fabrication processes, fabricated and characterized the devices and analyzed the data. M.D. synthesized the colloidal quantum dots materials and performed the initial material characterization. N.T. helped in the design and simulation of the metamaterial absorbers. R.B. and G.W. contributed to the ellipsometry measurements. R.B. contributed to UPS measurements. G.W. helped in the nano‐fabrication using e‐beam lithography for metamaterial absorbers. G.T.P. took the SEM images of the PMAs and helped in the experiments. M.D. helped in the automation of the optoelectronics setup and the optoelectronic characterization.

## Conflicts of Interest

The authors declare no conflicts of interest.

## Supporting information




**Supporting File**: adma73275‐sup‐0001‐SuppMat.docx.

## Data Availability

The data that support the findings of this study are available from the corresponding author upon reasonable request.
