# Supplementary material for: A Colloidal Quantum Dot Thermistor and Bolometer
Source: Adv Mater. 2026 May 4;38(32):e19385. doi: 10.1002/adma.202519385 (PMC13244801; doi:10.1002/adma.202519385)
Supplement: Supplementary file 1 — Supporting File: adma73275‐sup‐0001‐SuppMat.docx. [file ADMA-38-e19385-s001.docx]

**Supplementary Information**

**A Colloidal Quantum Dot Thermistor and Bolometer**

Gaurav Kumar^1^, Mariona Dalmases^1^, Nima Taghipour^1^, Rajesh Bera^1^, Guy L. Whitworth^1^, Goretti Torres Perez^1^, Miguel Dosil^1^, Gerasimos Konstantatos^1,2^*

^1^ICFO-Insitut de Ciencies Fotoniques, The Barcelona Institute of Science and Technology, Castelldefels, 08860 Barcelona, Spain.

^2^ICREA-Institució Catalana de Recerca i Estudiats Avançats, Lluis Companys 23, 08010 Barcelona, Spain.

*e-mail: Gerasimos.Konstantatos@icfo.eu

**
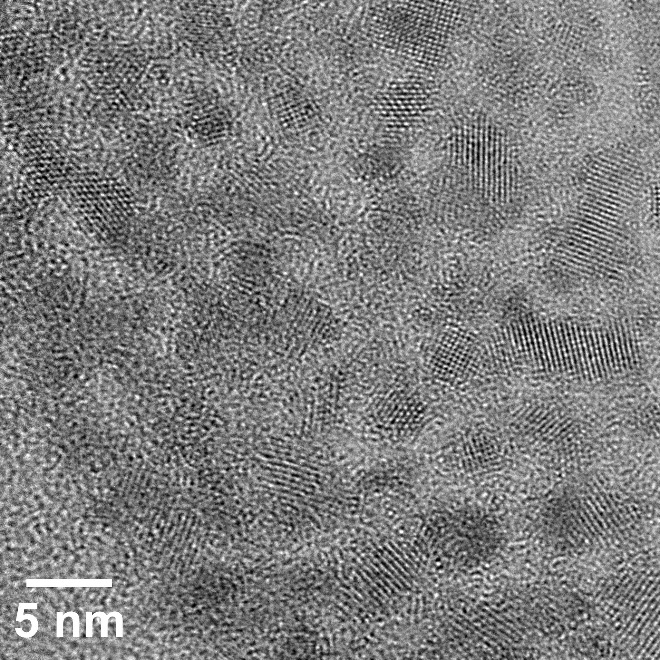

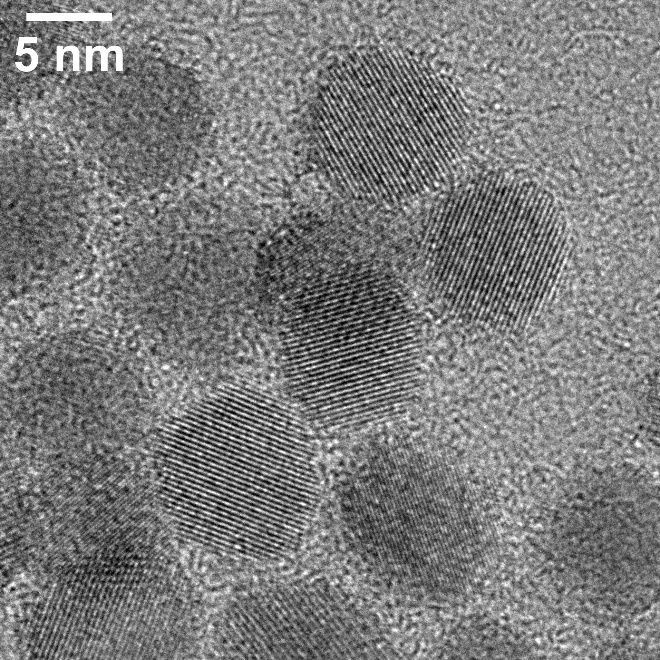
**

**Supplementary Figure 1.** TEM images of the PbS 850 nm (left) and PbS/PbSSe (right) CQDs

**
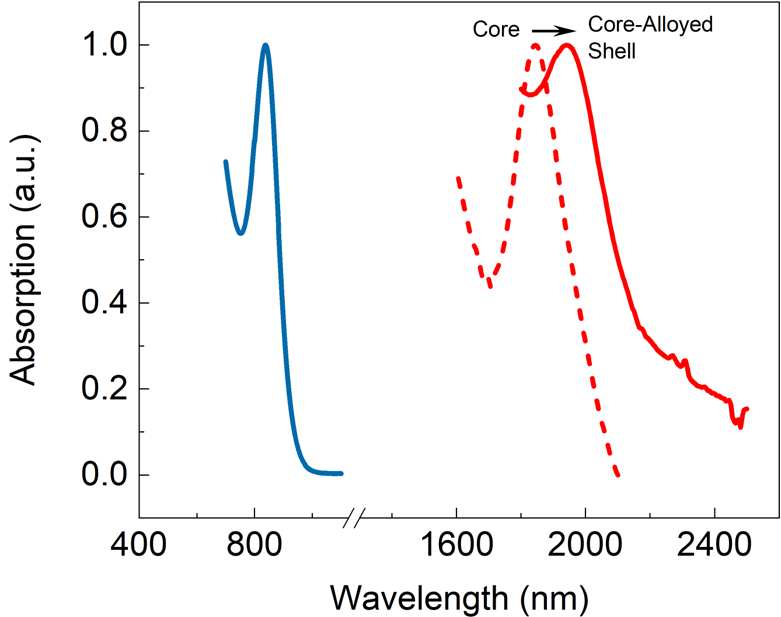
**

**Supplementary Figure 2.** Absorption data for PbS CQDs with exciton peak around 850 nm and PbS/PbSSe CQDs around 1950 nm.


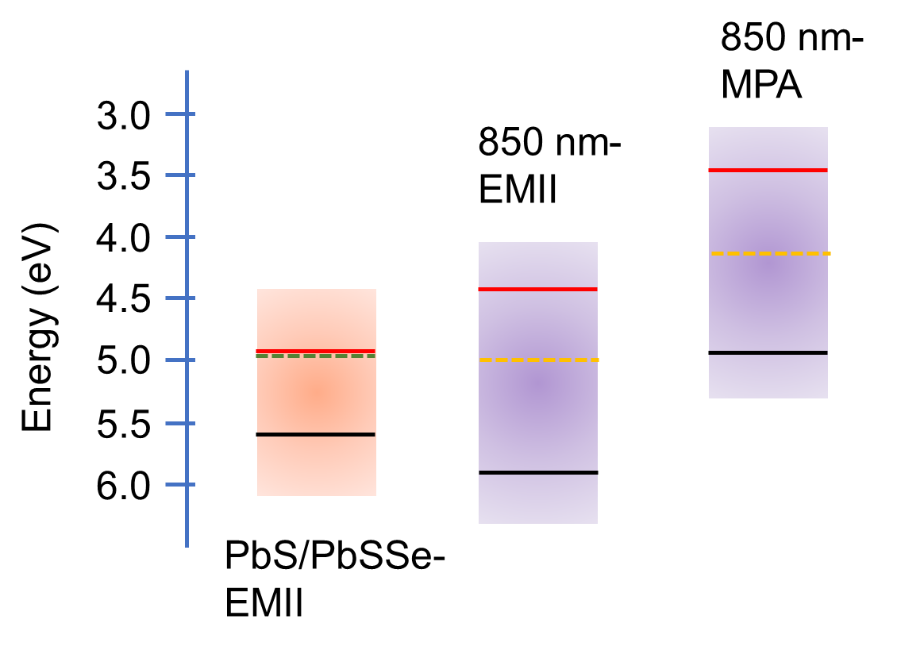


**Supplementary Figure 3:** Ultraviolet Photoelectron Spectroscopy (UPS) data showing the energy band positions of the CQDs with varying size and ligand treatment

**Raw data plots of UPS data used for generating supplementary Fig. 3:**


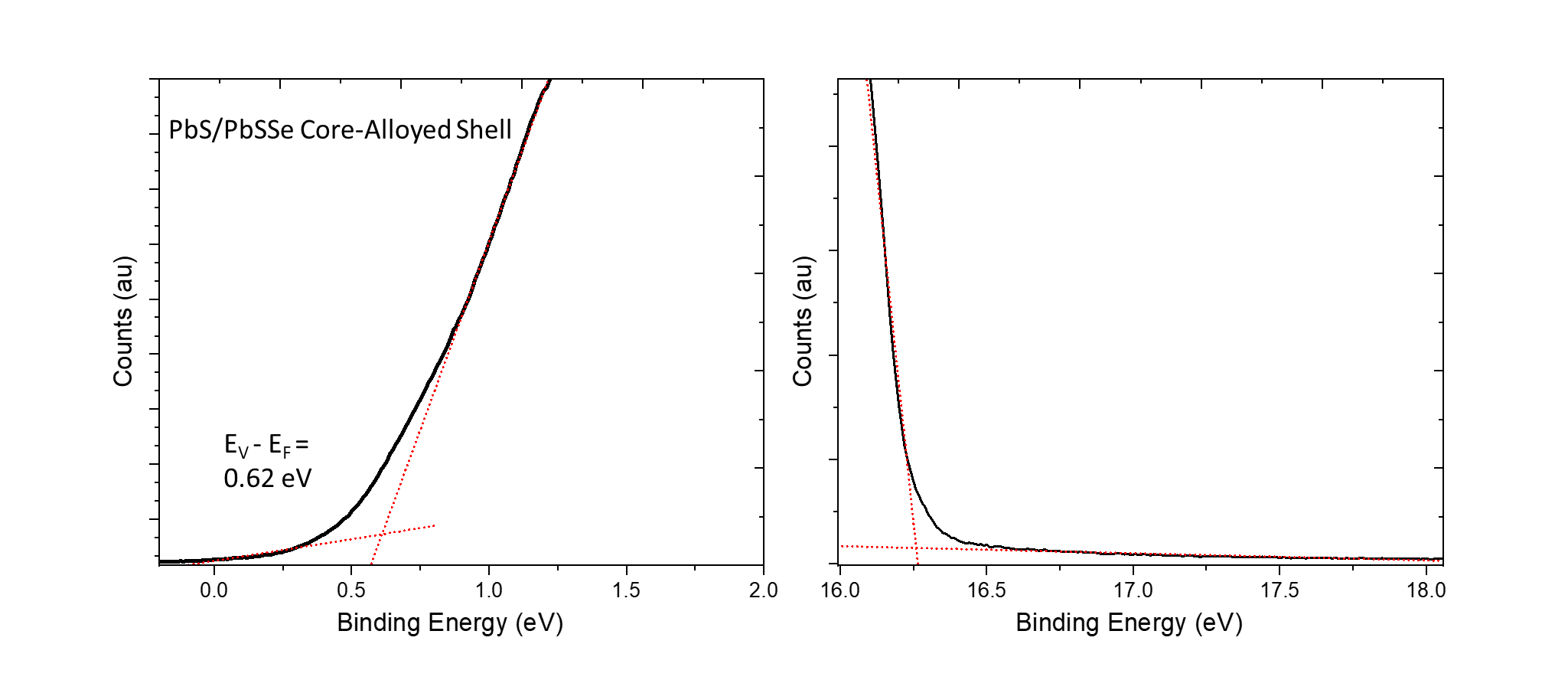


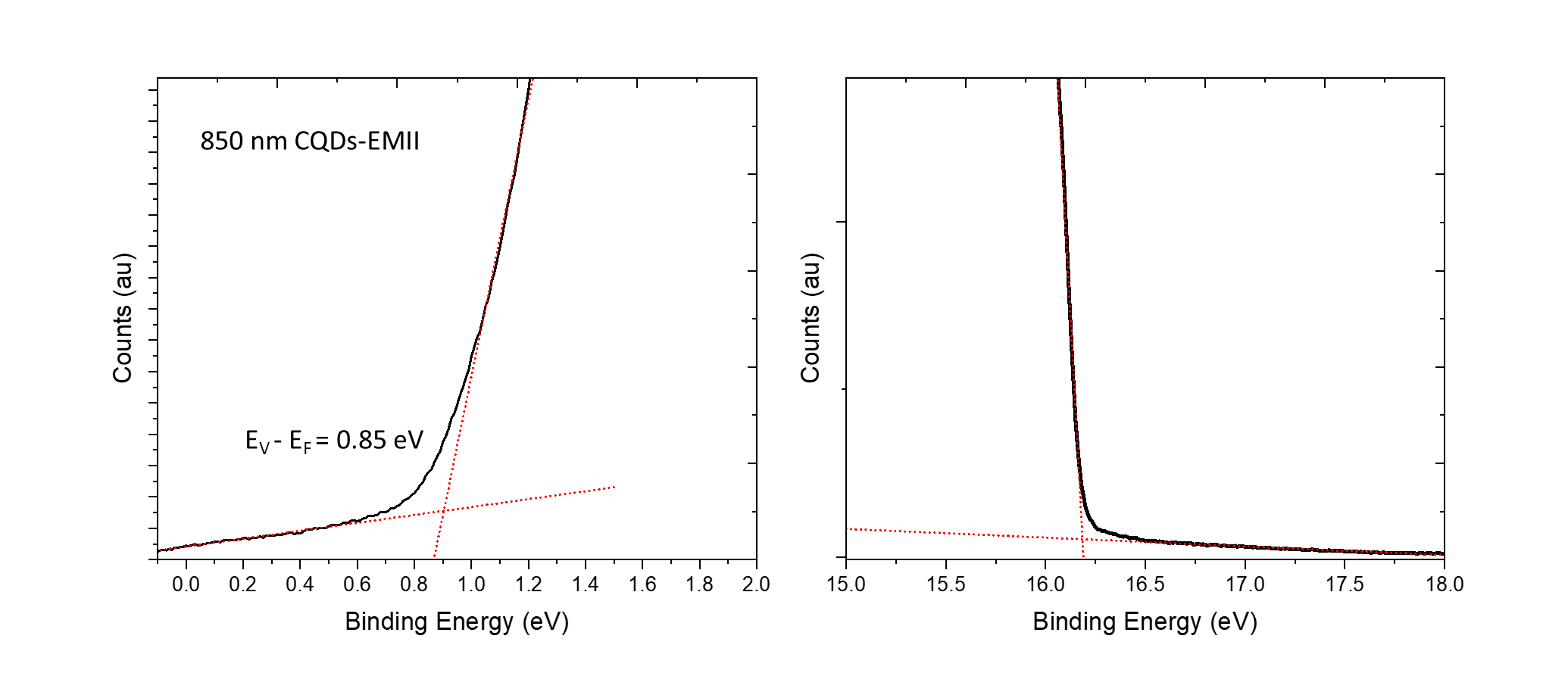


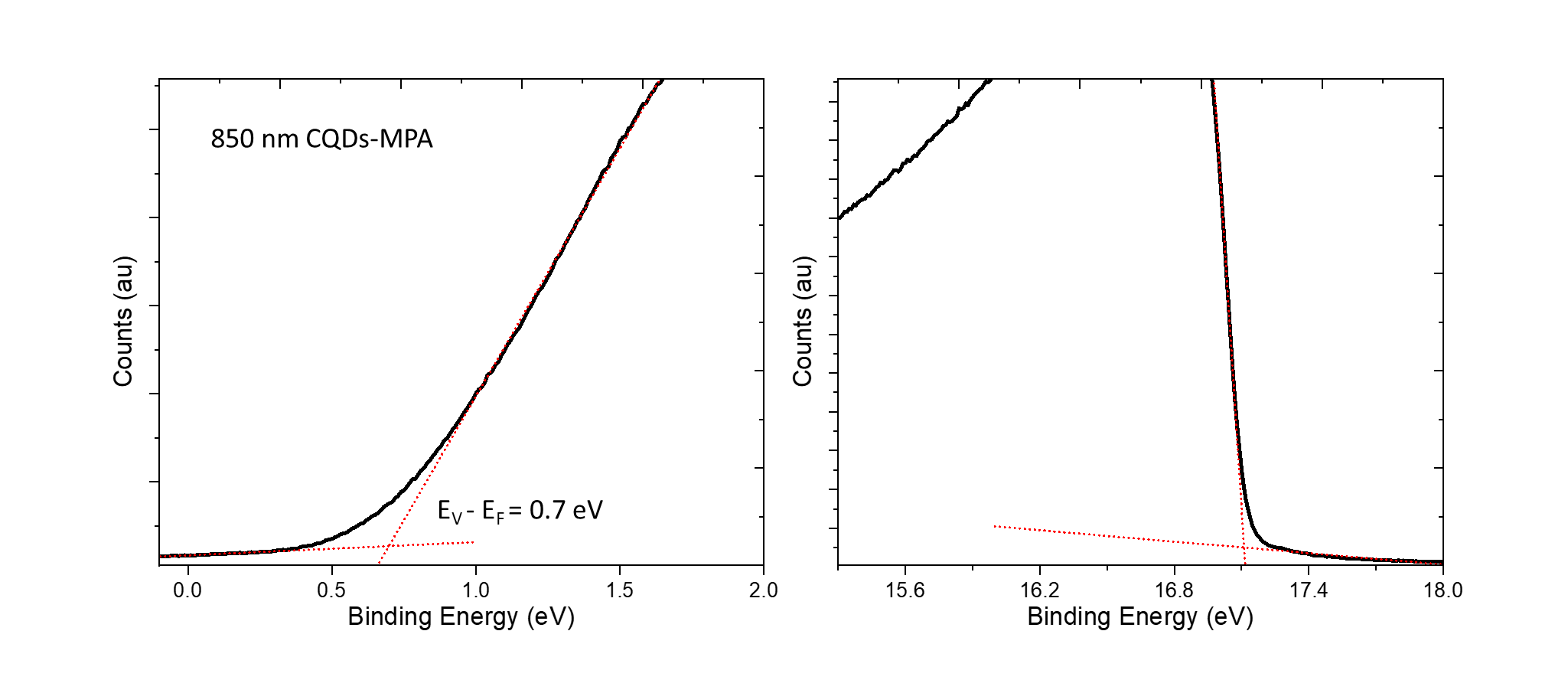


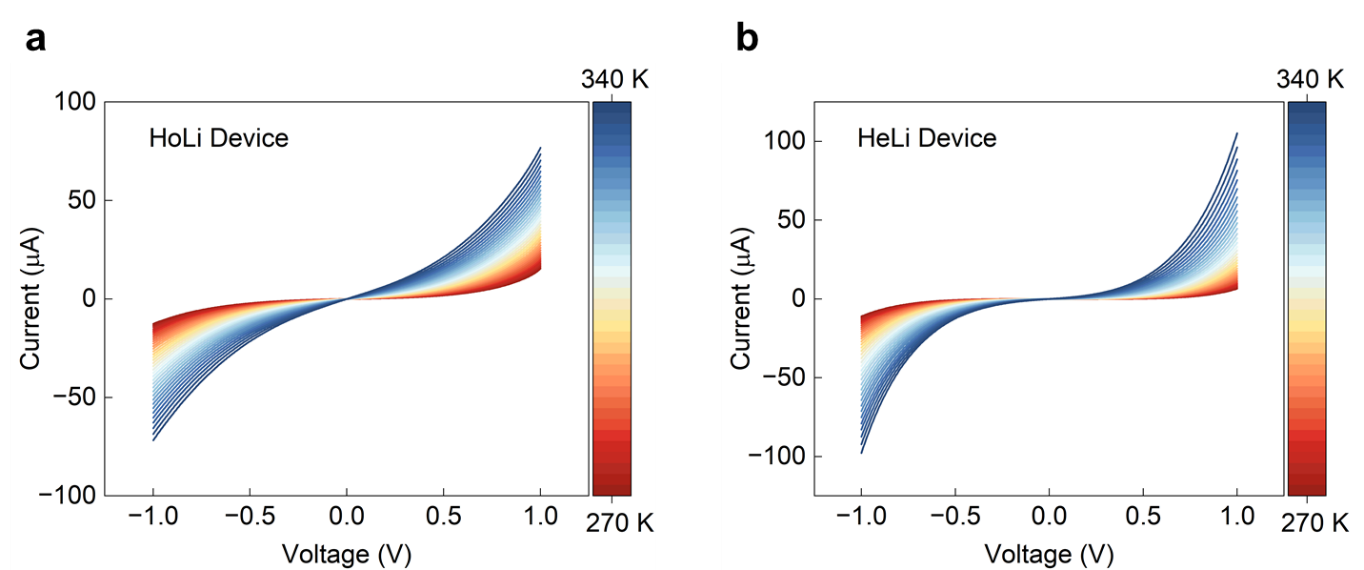


**Supplementary Figure 4: The temperature-dependent I-V characteristics of the QPBT. a**, Device with HoLi configuration., **b**, Device with HeLi configuration. The devices were kept in a cryostat under dark conditions and the I-V was recorded using a B1500A semiconductor analyzer from Keysight technologies after every ~12 mins.


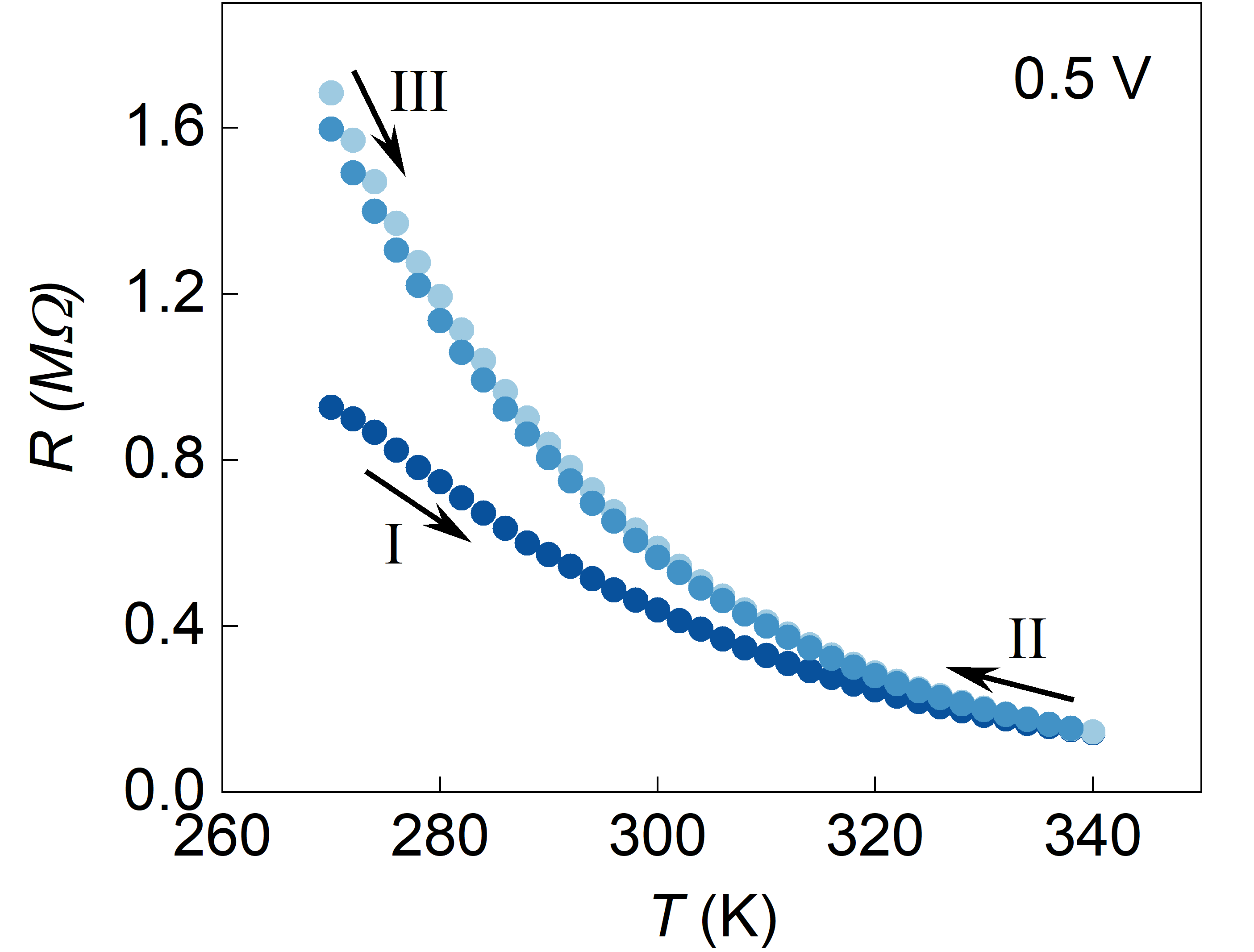


**Supplementary Figure 5:** The temperature dependent behaviour of a QDPBT sample under multiple cycles of heating and cooling.

**Supplementary Note 1: Fitting the *R* vs *T* data to extract *E_a_***

To confirm the existence of NNH type transport in our structure, as reported by many studies in the literature for the studied temperature range i.e. 270-340 K^[1-2]^, we first used the Arrhenius plot to confirm the absence of multiple slopes. As shown in the following Arrhenius plot, the measured data can be fitted with a R^2^ ~0.999. We also do not observe different slopes in the Arrhenius plot, which also implies that NNH is truly the dominating mechanism in the studies temperature range.


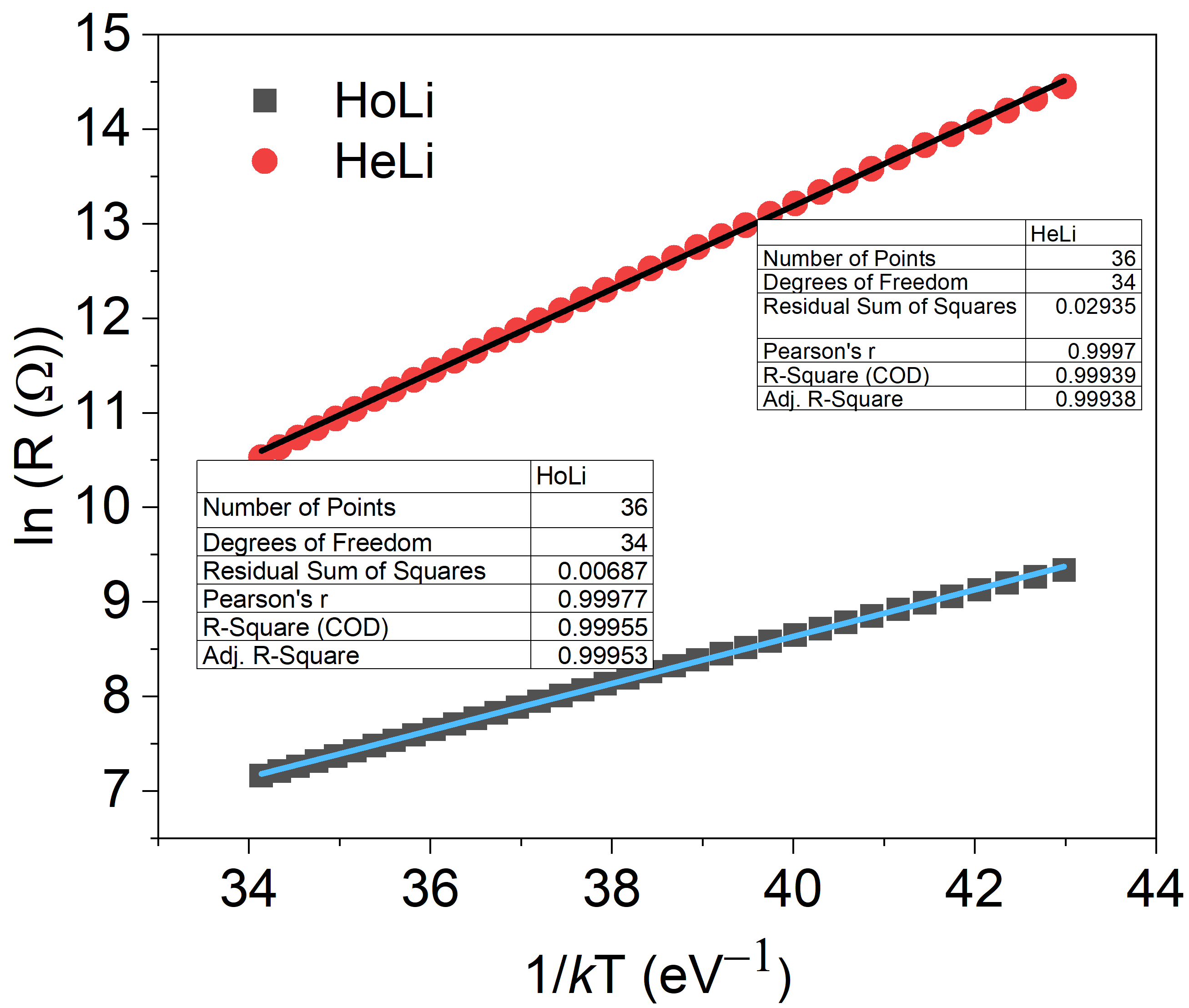


**Supplementary Figure 6:** Arrhenius plot showing the fit for *R* vs *T* data

To further confirm our assumption, we have also compared the fitting of R vs T data with the general VRH equation given by:

$$R=R_{o}exp\left[ \left( \frac{T_{o}}{T} \right)^{v} \right]$$

where, *v* is either ¼ for Mott-VRH or ½ for Efros-Shklovskii (ES-VRH). The plots are shown below for both type of VRH for HoLi (a, b, and c) and HeLi (d, e and f). The corresponding R^2^ values are also provided to ascertain the goodness of fit in the table following the figures.


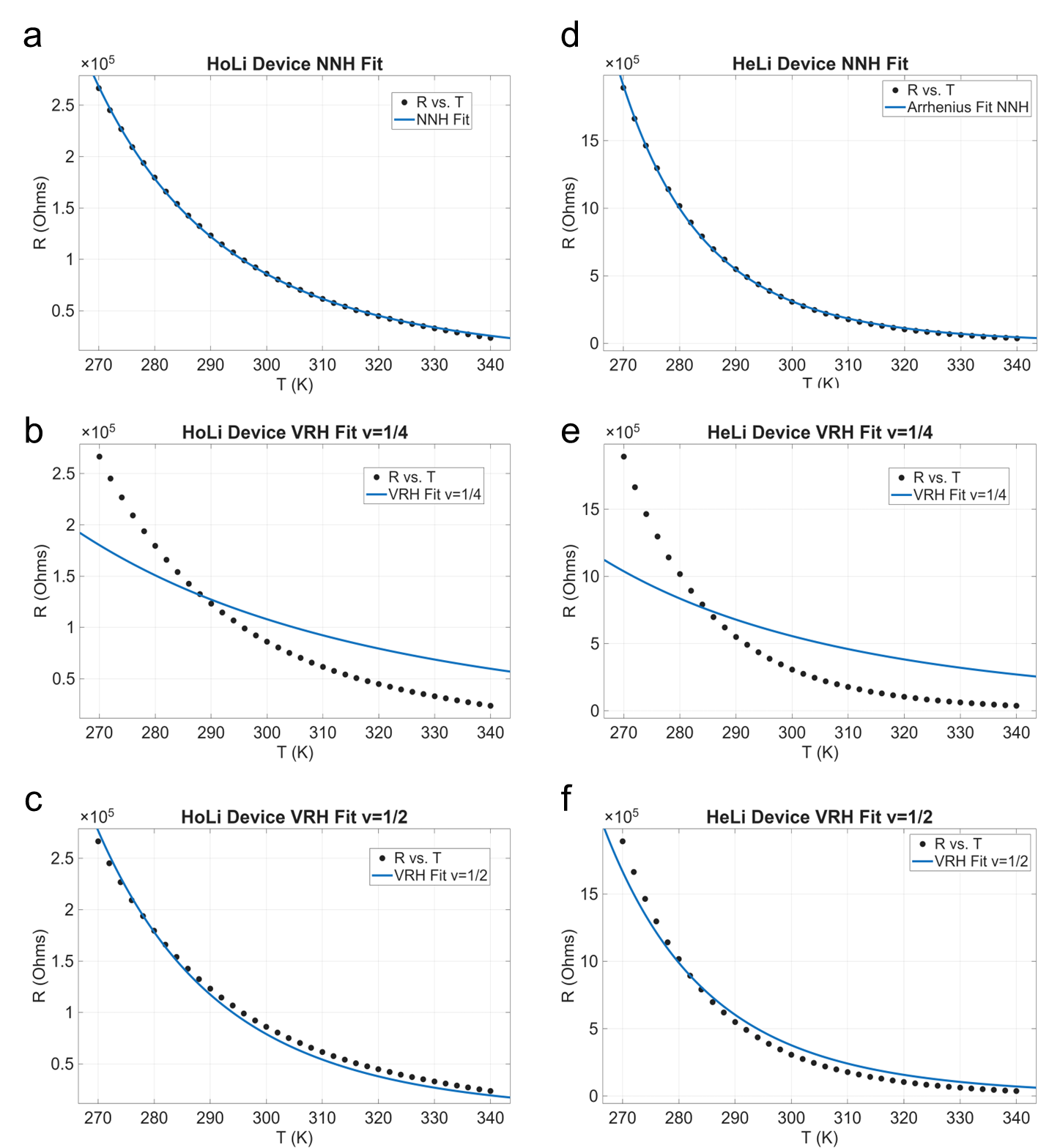


**Supplementary Figure 7:** Fitting of *R* vs *T* data to confirm the transport mechanism NNH over VRH.

**Supplementary Table 1: R^2^ values for the above plots**

| **Fit Type** | **HoLi Device** | **HeLi Device** |
| --- | --- | --- |
| NNH | 0.9998 | 0.9997 |
| Mott-VRH, v=1/4 | 0.73 | 0.6177 |
| ES-VRH, v=1/2 | 0.9752 | 0.9731 |

It is evident that the best fit is provided by the Arrhenius NNH, therefore confirming the charge conduction mechanism in the proposed structure to be of NNH origin. Moreover, since the Arrhenius plot data exhibit a single linear activation slope across the entire temperature range, the transport mechanism is dominated by thermally activated NNH rather than VRH.

[1] E. Romero, M. Drndic, *Phys. Rev. Lett.* **2005**, 95, 156801.

[2] K. Whitham, J. Yang, B. H. Savitzky, L. F. Kourkoutis, F. Wise, T. Hanrath, *Nat. Mater.* **2016**, 15, 557.


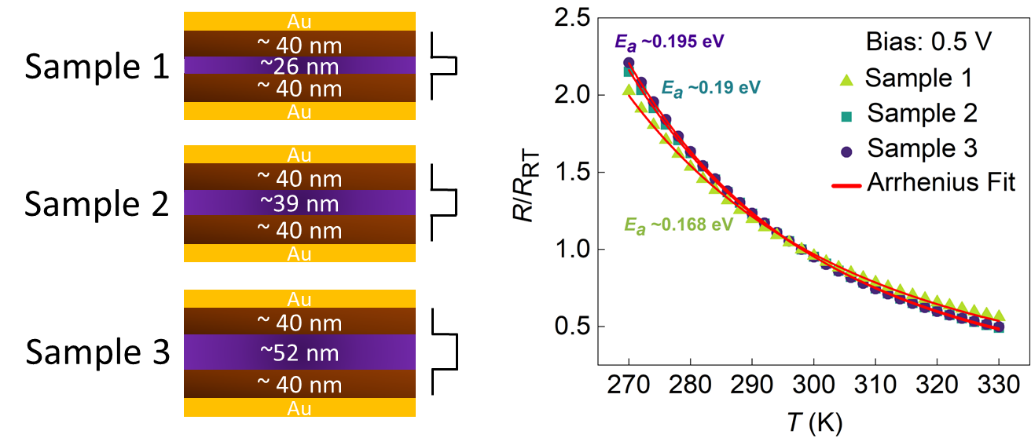


**Supplementary Figure ­8: Effect of varying the thickness of PB layer on the QDPBT performance. T**he performance of QDPBT with HoLi configuration and QDs with exciton peak at 850 nm as PB layers QDs, in a single potential barrier (SPB) geometry were used for this study.

**
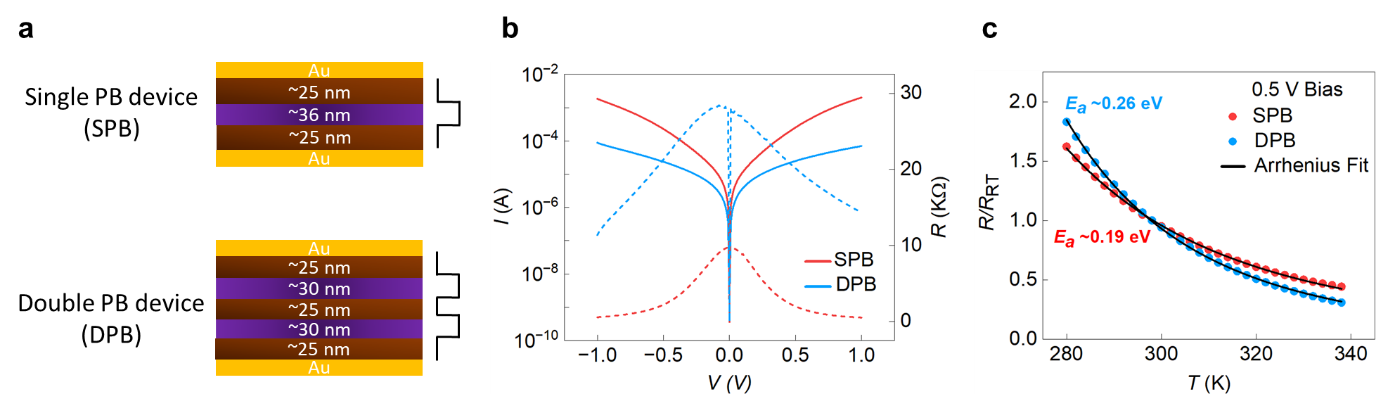
**

**Supplementary Figure ­­9: A comparison of the performance of QDPBT with single PB (SPB) and double PB (DPB) layers with HoLi configuration, using CQDs having an exciton peak ~850 nm as the PB layer QDs. a,** Schematic illustrating the SPB and DPB configurations, with approximate layer thickness as determined by contact profilometry. **b,** Room-temperature *I-V* characteristics demonstrating an increase in the device resistance for the DPB sample. The solid lines represent the *I-V* data and the dotted plots represent the *R-V* data. **c,** The variation of resistance with temperature for both the samples, fitted with Arrhenius equation, showing a stronger temperature dependence for DPB device as compared to SPB, where *E_a_* is ~0.19 and ~0.26 eV for SPB and DPB devices, respectively.

**Effect of Varying PB layer QDs Size:**

**S1: smaller CQDs with exciton peak at ~700 nm as PB**

**S2: smaller CQDs with exciton peak at ~850 nm as PB**

**S3: smaller CQDs with exciton peak at ~1100 nm as PB**


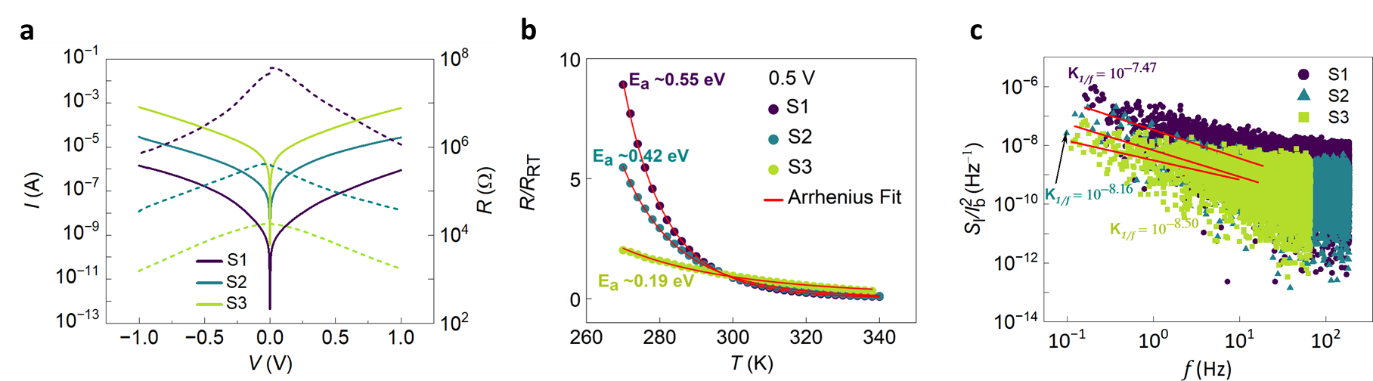


**Supplementary Figure 10: The effect of varying the size of PB-layer-CQDs on the thermistor performance of QPBT in HeLi configuration**. **a,** Room-temperature *I-V* characteristics demonstrating an increase in the device resistance with a decrease in size of PB-layer-CQDs. The solid lines represent the *I-V* data and the dotted plots represent the *R-V* data. **b,** The variation of QPBT resistance with temperature, fitted with Arrhenius equation, showing the strongest dependence for device S1 with smallest sized PB-layer-CQDs and weakest for device S3 with largest sized PB-layer-CQDs. **c,** The change in the normalized noise of the devices at a representative voltage of 0.5 V.

**
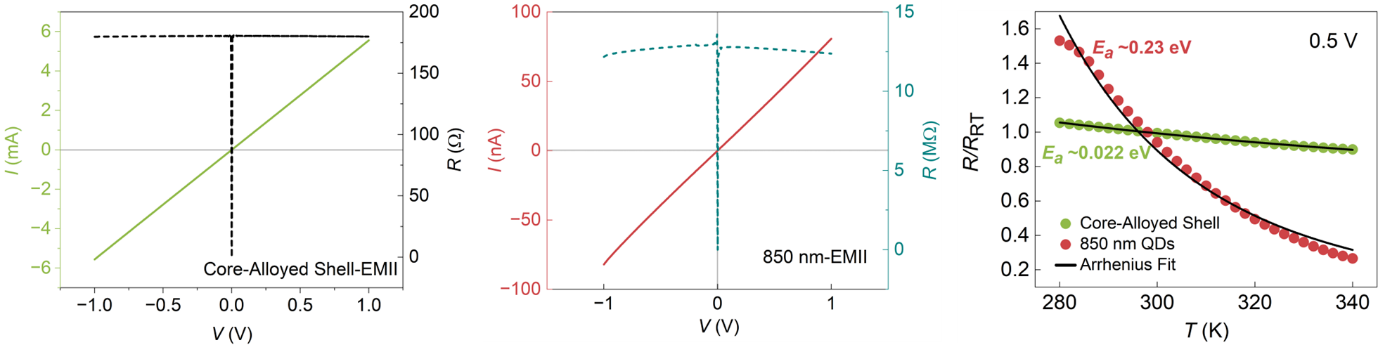
**

**Supplementary Figure 11:** Electrical characterization of single-type QD films, (left) Core-alloyed shell CQDs treated with EMII, (center) 850 nm CQDs treated with EMII. (right) Resistance (normalized to the resistance at room temperature) as a function of temperature for the two types of devices, and the extracted activation energies for the two classes of CQDs.


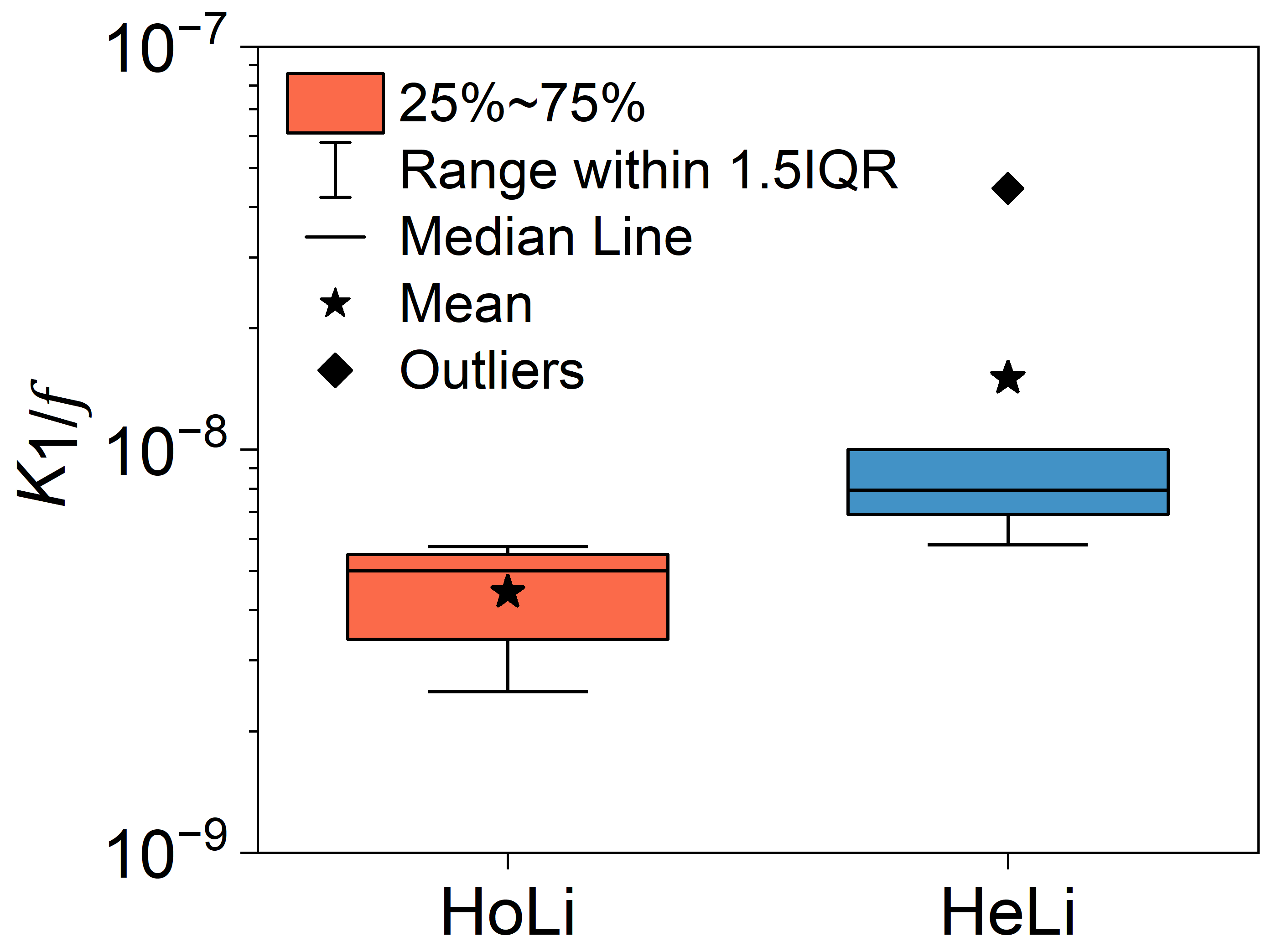


**Supplementary Figure 12:** A box plot showing the statistics of noise parameter among five devices of DPB HoLi and HeLi configuration, with 850 nm QDs as PB layer.

**Raw data plots used to generate supplementary Fig. 11:**

**HoLi Devices:**


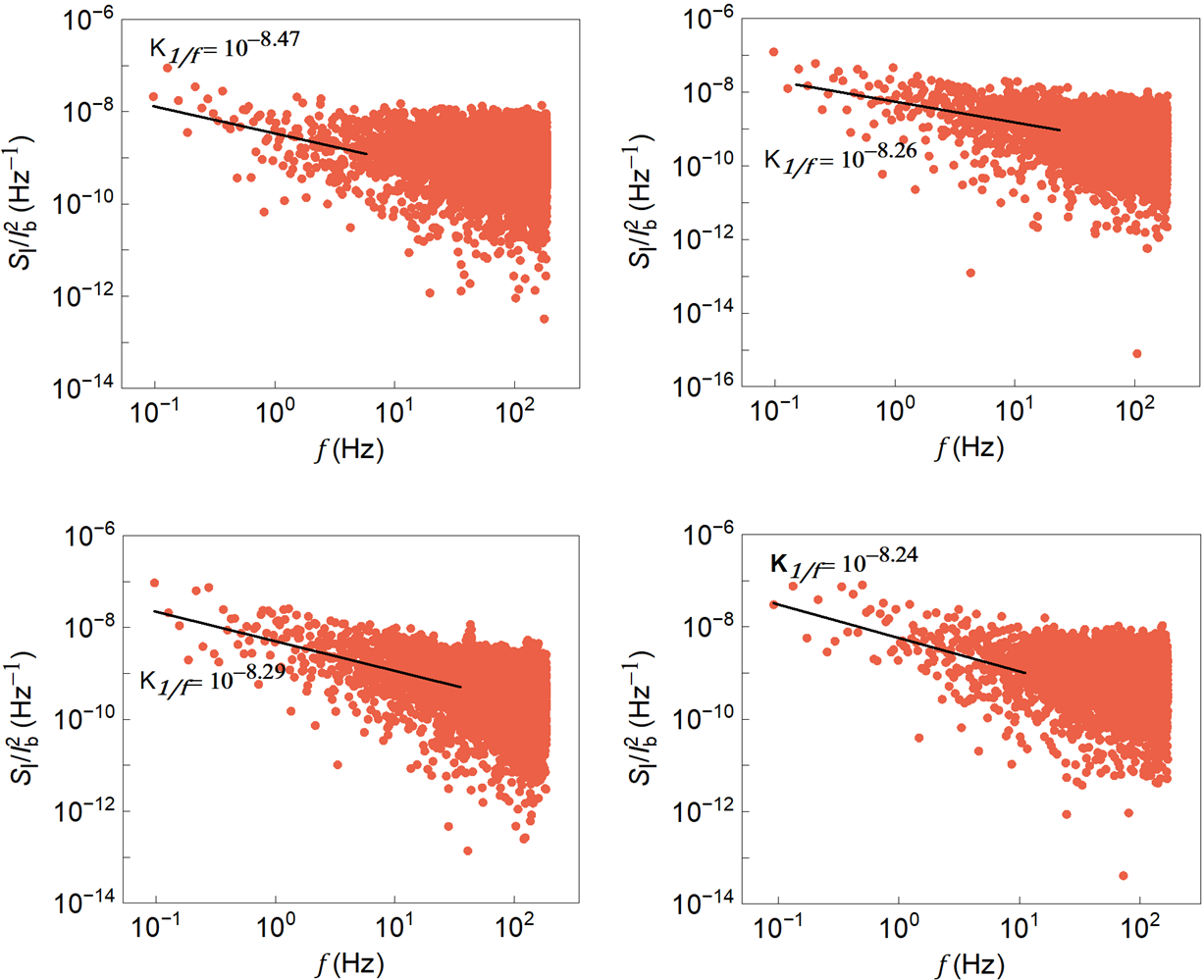


**HeLi Devices:**

**
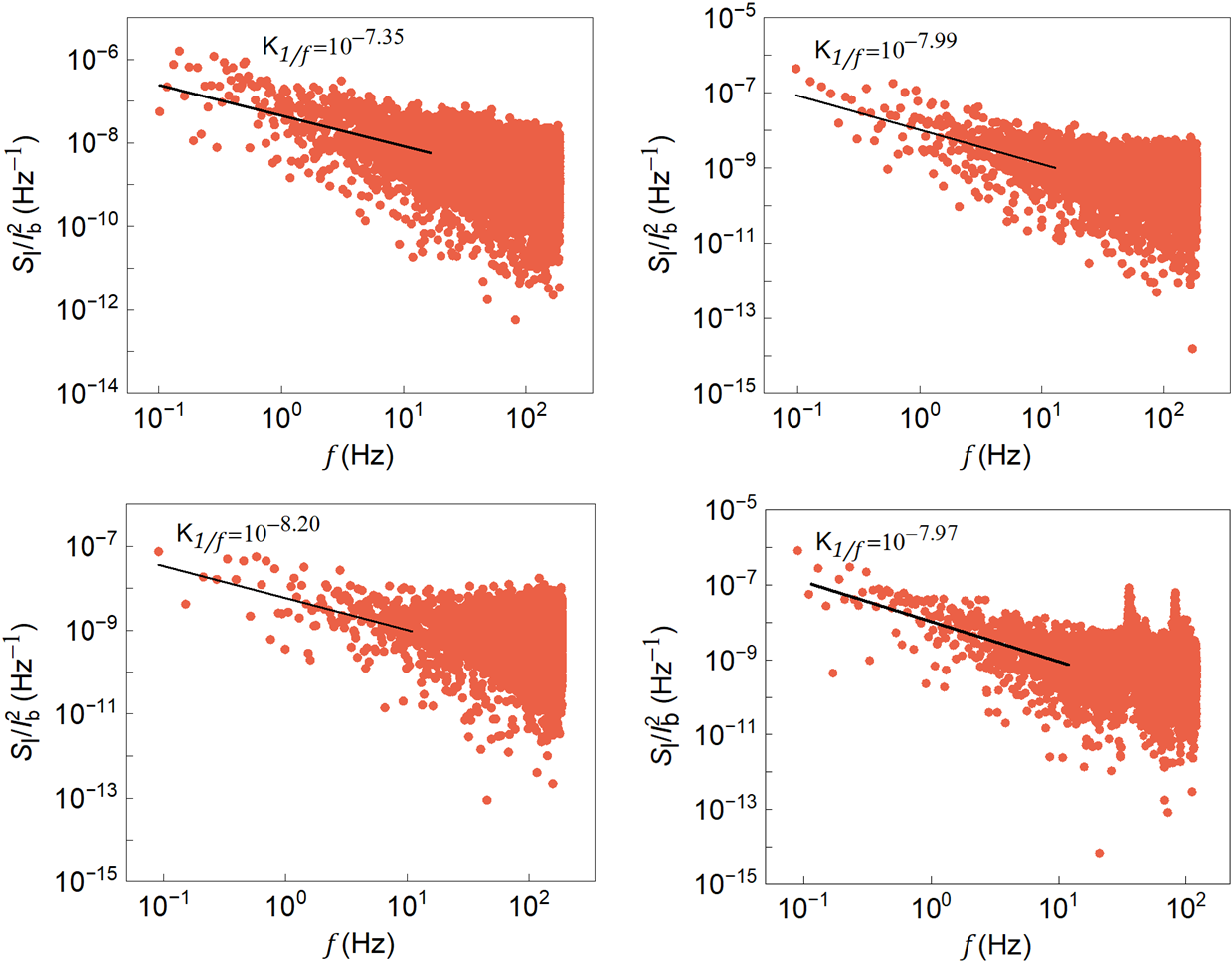
**

**Bias Dependence of K*_1/_*_f_ noise:**

**
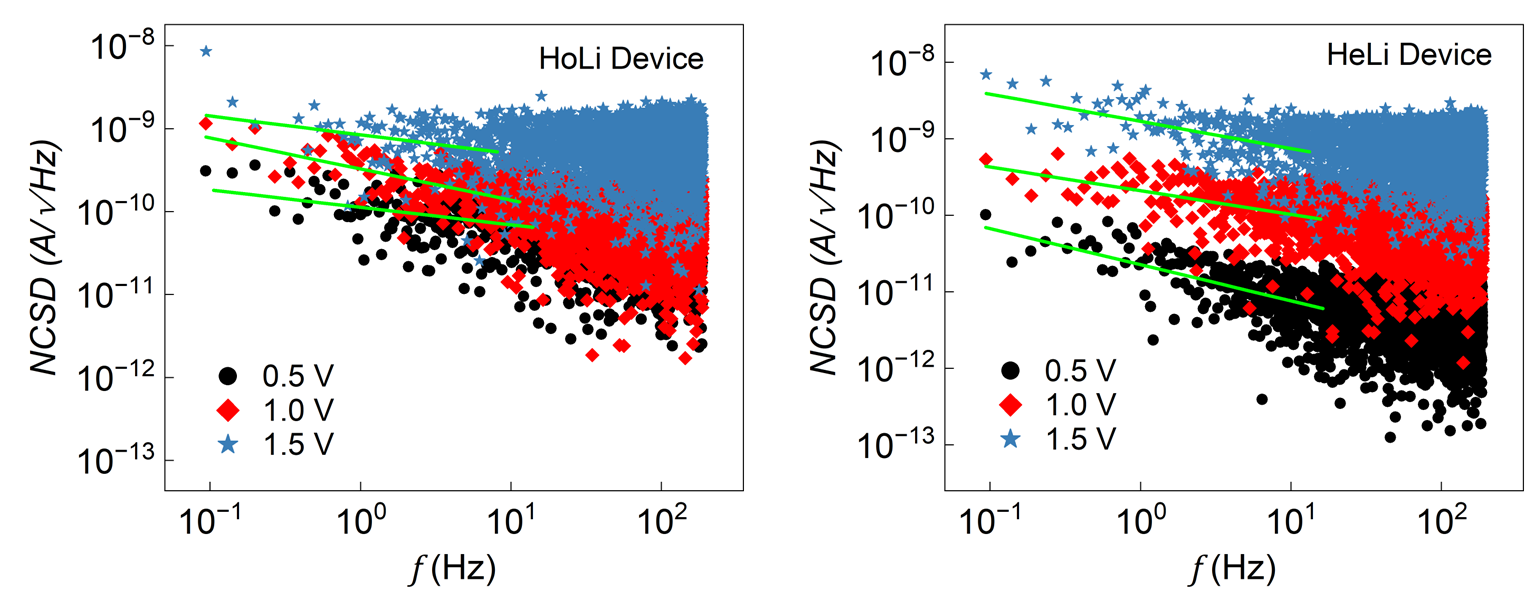
**

**Supplementary Figure 13:** The evolution of noise with the bias for both DPB HoLi and HeLi configuration devices, with 850 nm QDs as PB layer.

**Supplementary Table 2:** Value of noise at 1 Hz from log-log slope from the supplementary Fig. 12

| **Bias** | **HoLi Device** | **HeLi Device** |
| --- | --- | --- |
| 0.5 V | 1.12e-10 | 2.23e-11 |
| 1.0 V | 3.24e-10 | 2.08e-10 |
| 1.5 V | 8.48e-10 | 1.69e-9 |

**
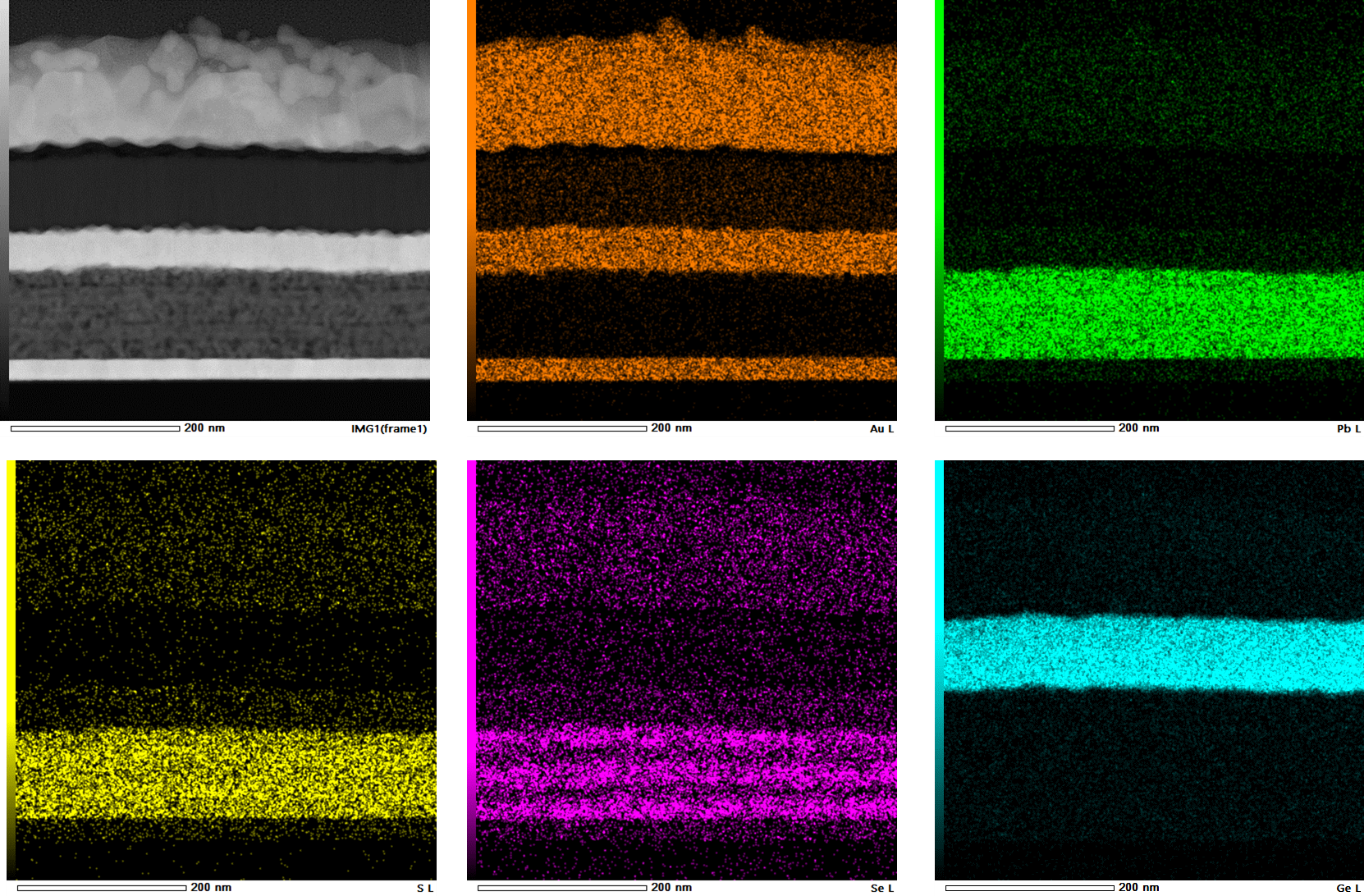
**

**Supplementary Figure 14.** Cross-sectional microscopy and spectroscopy analysis of the complete bolometer device structure.


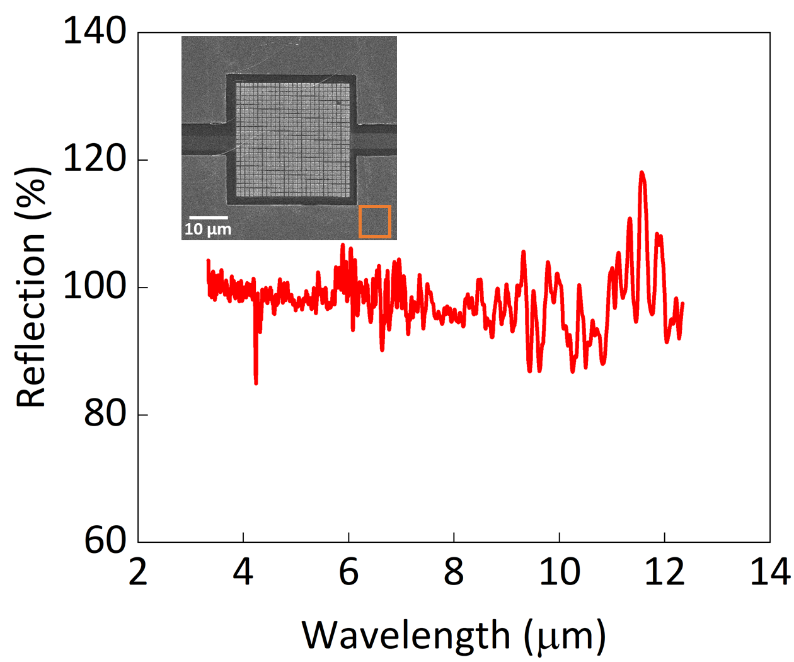


**Supplementary Figure 15:** FTIR measurement over the indicated area in SEM (inset) on Au window around the bolometer pixel to reflect the excess light falling on the surroundings.

**Supplementary Note 2: Estimation of *n-k* values for e-beam deposited Ge film**

**
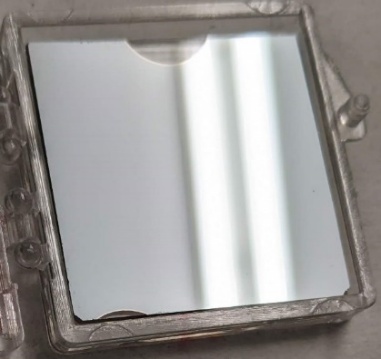
**

**Supplementary Figure 16:** An image of the e-beam evaporated Ge on borosilicate glass.

The e-beam deposited Ge film was characterized for its refractive index *(n(λ))* and extinction coefficient *(k(λ))* (as described in methods). The substrate chosen here was glass. The resulting *n(λ)* and *k(λ)* values of the material were then plotted and used to parametrize the optical model of the device.^[1-2]^


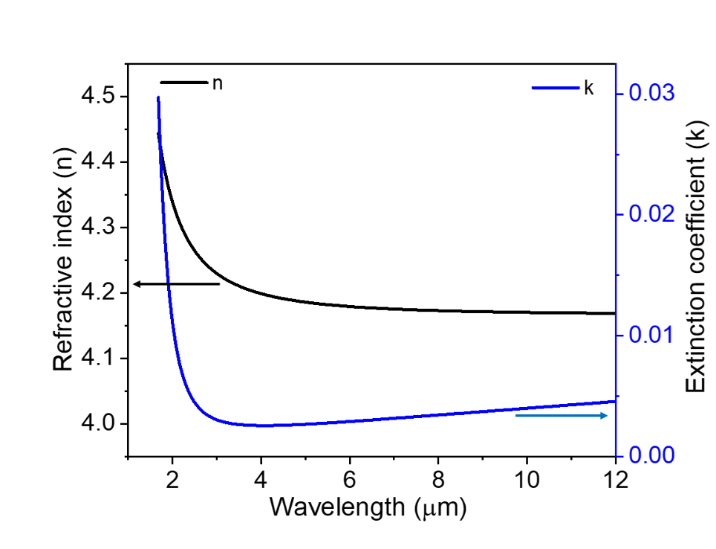


**Supplementary Figure 17:** Evaluated Refractive index (black) and Extinction coefficient (blue) of the Ge films from ellipsometry.

[1] J. H. Burnett, E. C. Benck, S. G. Kaplan, E. Stover, A. Phenis, *Appl. Opt.* **2020**, 59, 3985.

[2] T. Amotchkina, M. Trubetskov, D. Hahner, V. Pervak, *Appl. Opt.* **2020**, 59, A40.

**
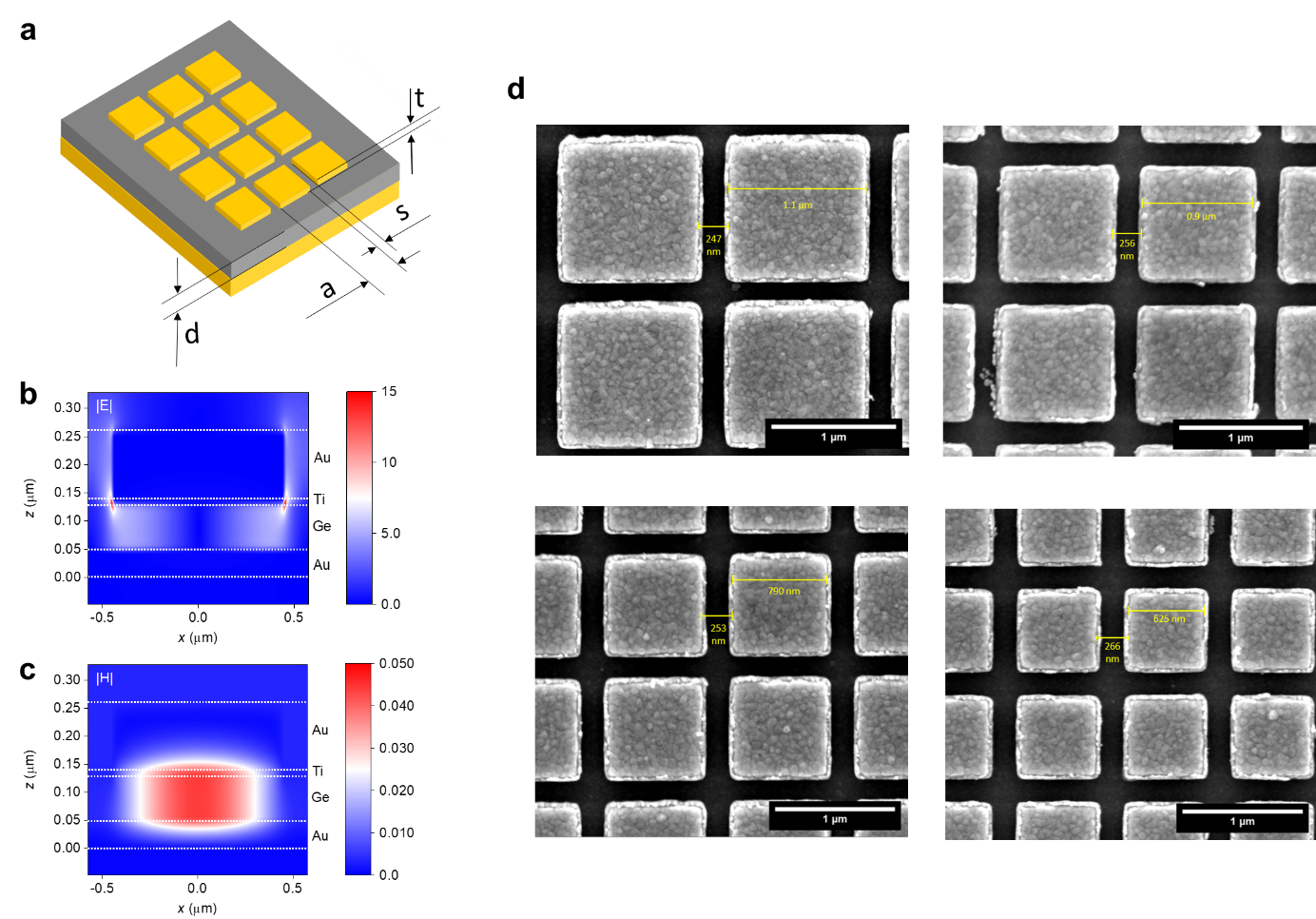
**

**Supplementary Figure 18: Ge-based PMA**. a, The geometry of metamaterial structure. b, and c, Electric-field (|E|) and Magnetic Field (|H|) profiles of the PMA at the resonant frequency simulated using Lumerical FDTD. d, The fabricated metamaterial structures with varying sizes to demonstrate multispectral sensing.

**Supplementary Note 3: Working of the Ge-based PMA**

**Supplementary Figure 19:** Reflection Spectra of the PMA. A comparison between the simulated and measured reflection spectra of the PMA also with the measured spectra on the device after Au window formation.

Supplementary Fig. 19 presents a detailed comparison of the reflectance as a function of wavelength for the device structure, featuring the simulated reflectance spectrum (red solid line), modeled based on the configuration shown in supplementary Figure 7, and the experimentally measured reflectance from the fabricated device (blue solid line). In both the simulation and experimental setup, the incident light is directed at normal incidence to ensure consistent optical boundary conditions. The results demonstrate good agreement between the measured and simulated reflectance spectra, validating the accuracy of the optical model. The observed resonance mode originates from the synergistic interplay between localized surface plasmon resonance (LSPR) and Fabry-Perot modes. The LSPR is excited at the interface between the Au-based metamaterial structure and the Ge layer, where sub-micron patterning enhances the near-field confinement at the metal-dielectric interface. Simultaneously, the Fabry-Perot mode arises from the confinement of light within the Ge layer, enabled by the reflective Au layer positioned between the CQDs thermistor and the Ge layers. This Au layer acts as a highly reflective mirror in the operating region, forming an optical cavity between the metamaterial pixel and the Au back reflector, supporting resonance mode within the Ge layer (distinct dips in the reflectance spectrum on the shorter wavelengths). It is worth noting that the addition of an Au window surrounding the metamaterial pixel (30 μm × 30 μm) leads to a noticeable blue shift in the resonance, shifting from approximately 10 μm to around 9 μm. This spectral shift can be attributed to several underlying mechanisms. Primarily, the proximity of the Au layer—positioned at a lateral distance of ~1.5 μm from the active pixel—modifies the boundary conditions of the surrounding unpatterned region. The Au layer significantly enhances the reflection of incident light from the adjacent CQD layers, thereby amplifying the contribution of the Fabry–Perot resonance within the optical cavity. The increased reflectivity significantly strengthens the constructive interference conditions necessary for this resonance, particularly in the shorter wavelength regime. As a result, the hybrid resonance mode becomes increasingly dominated by the Fabry–Perot component, which intrinsically supports resonances at shorter wavelengths. This enhanced the effect of Fabry–Perot, combined with the modified optical feedback due to the surrounding Au window, collectively contributes to the observed blue shift in the resonance wavelength.


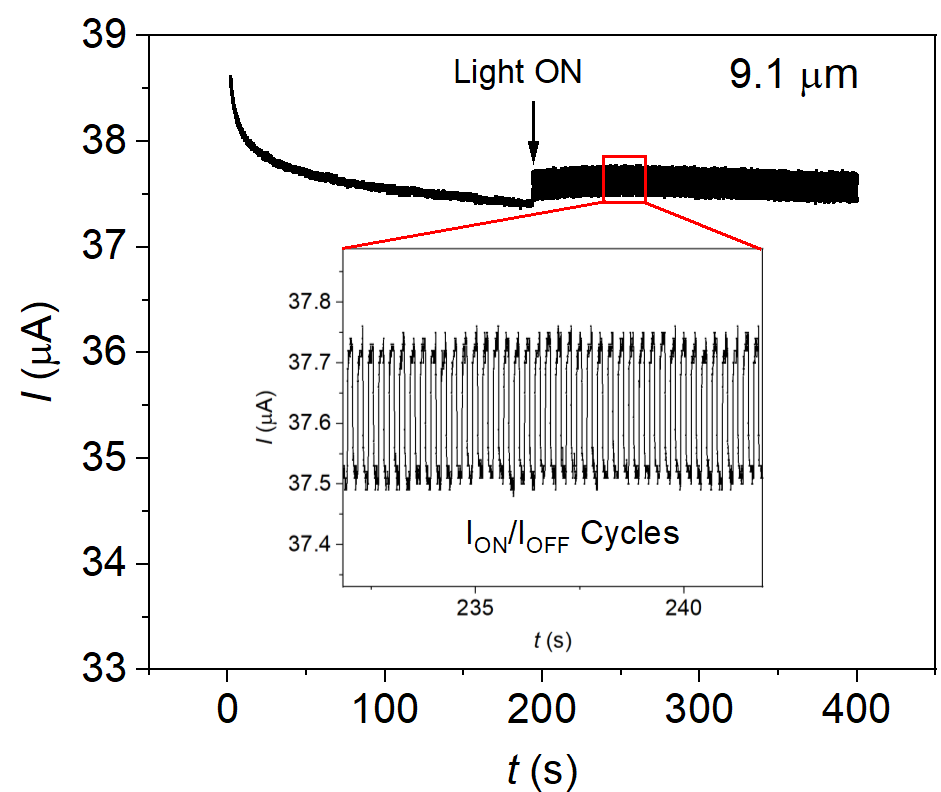


**Supplementary Figure 20:** Plot showing the device current drift stabilizing around ~200s, alongwith long light exposure with optical chopping, to show the stability of the device response.

**Supplementary Note 4: Quantum Cascade Laser (QCL) beam spot and power estimation:**

To measure the spot size of the QCL, a pyroelectric camera from mks-Ophir photonics (Pyrocam III HR Laser Beam Profiler) was utilized. The shape of the QCL beam was confirmed multiple times by placing the laser beam on different spots around the center of the parabolic Au mirror.

**
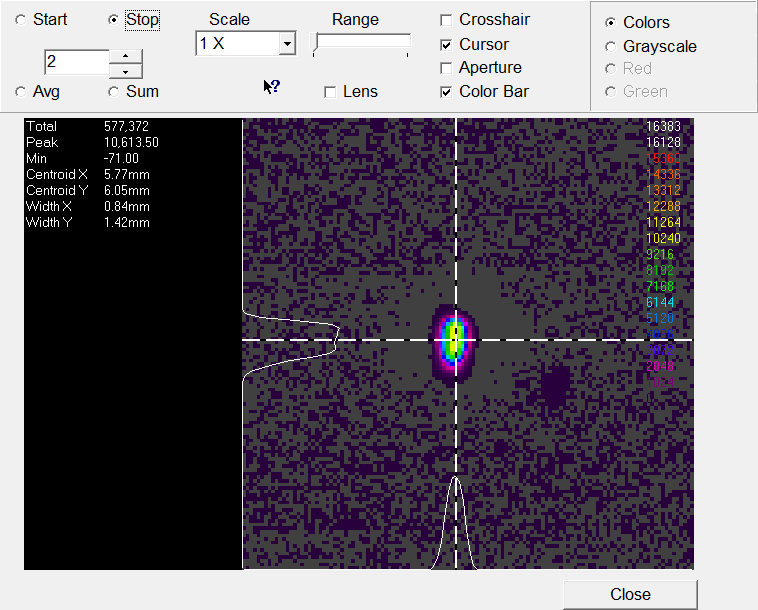
**

**Supplementary Figure 21:** QCL laser spot measured using a pyroelectric camera

**Power estimation through the Au window:**

The irradiance of a Gaussian beam can be described by:

$$I \left( r \right)= I_{0}e^{\frac{-2r^{2}}{w^{2}}}=\frac{2P}{\pi w^{2}}e^{\frac{-2r^{2}}{w^{2}}}$$

Where, r is the radial distance from the central axis of the beam, *w* is the radius at which the beam intensity fall to 1/e^2^ of their axial values, *I_0_* is the peak intensity at the center of the beam, and *P* is the total power of the beam.

Integrating over an area with a radius of *r*, the total power contained in an area of radius *r* can be given by,

$$P \left( r \right)=P_{0}[1-e^{\frac{-2r^{2}}{w^{2}}}]$$

For an elliptical beam,

$$I \left( x,y \right)= I_{0}exp\left[ -2\left( \frac{x^{2}}{w_{x}^{2}}+\frac{y^{2}}{w_{y}^{2}} \right) \right]$$

where, *I*_0_ is the peak intensity, *w_x_* and *w_y_* are the beam radii along the x and y axes, respectively.

The power transmitted through an aperture can thus be calculated by integrating the above equation:

$$P_{Aperture}=\int_{-a}^{a} \int_{-b}^{b} I \left( x,y \right) dx dy=I_{0}\int_{-a}^{a} \int_{-b}^{b} exp\left[ -2\left( \frac{x^{2}}{w_{x}^{2}}+\frac{y^{2}}{w_{y}^{2}} \right) \right]dx dy$$

$$=I_{0}\left( \int_{-a}^{a} exp\left[ \frac{-2x^{2}}{w_{x}^{2}} \right]dx \right)\left( \int_{-b}^{b} exp\left[ \frac{-2y^{2}}{w_{y}^{2}} \right]dy \right)$$

Where, *a* and *b* are the half widths of a rectangular aperture along the x and y axis, respectively.

The two integrals involve the Gaussian function, for x and y axis. Hence, these integrals can be expressed in the form of error functions, $\mathrm{erf} \left( x \right)= \left( 2/\sqrt{\pi} \right)\int_{0}^{x} e^{{-t}^{2}}dt$, so that:

$$\int_{-a}^{a} exp\left[ \frac{-2x^{2}}{w_{x}^{2}} \right]dx=\frac{w_{x}\sqrt{\pi}}{\sqrt{2}}erf\left( \frac{\sqrt{2}a}{w_{x}} \right)$$

and,

$$\int_{-b}^{b} exp\left[ \frac{-2y^{2}}{w_{y}^{2}} \right]dy=\frac{w_{y}\sqrt{\pi}}{\sqrt{2}}erf\left( \frac{\sqrt{2}b}{w_{y}} \right)$$

Thus,

$$P_{Aperture}=I_{0} \left( \frac{w_{x}\sqrt{\pi}}{\sqrt{2}}erf\left( \frac{\sqrt{2}a}{w_{x}} \right) \right)\left( \frac{w_{y}\sqrt{\pi}}{\sqrt{2}}erf\left( \frac{\sqrt{2}b}{w_{y}} \right) \right)$$

Moreover, for an elliptical laser beam:

$$P_{Total}=\frac{\pi I_{0}w_{x}w_{y}}{2}$$

Thus, the fraction of power transmitted is given by:

$$\frac{P_{Aperture}}{P_{Total}}=erf\left( \frac{\sqrt{2}a}{w_{x}} \right) erf\left( \frac{\sqrt{2}b}{w_{y}} \right)$$

The above equation implies that the fraction of power transmitted through an aperture can be approximated by the product of the error functions in each dimension, where a and b are the half widths of the aperture in x and y dimensions, respectively.

For an aperture of the size of thermistor pixel 30 µm × 30 µm;

$$a=b=15 \mu m$$

And, elliptical beam dimensions (1/e^2^ diameters):

X axis: *d_x_* = 0.84mm, so the radius $w_{x}$= 0.42 mm= 420 $\mu m$

Y axis: *dy* = 1.42 mm, so the radius $w_{y}$= 0.71 mm= 710 $\mu m$

$$\frac{P_{Aperture}}{P_{Total}}=erf\left( \frac{\sqrt{2}\times15}{420} \right) erf\left( \frac{\sqrt{2}\times15}{710} \right)$$

$$=erf\left( 0.05051 \right) erf\left( 0.02988 \right)$$

$$\frac{\boldsymbol{P}_{\boldsymbol{Aperture}}}{\boldsymbol{P}_{\boldsymbol{Total}}}\boldsymbol{=}0.05694\times0.03370\boldsymbol{=0.00192}$$

***
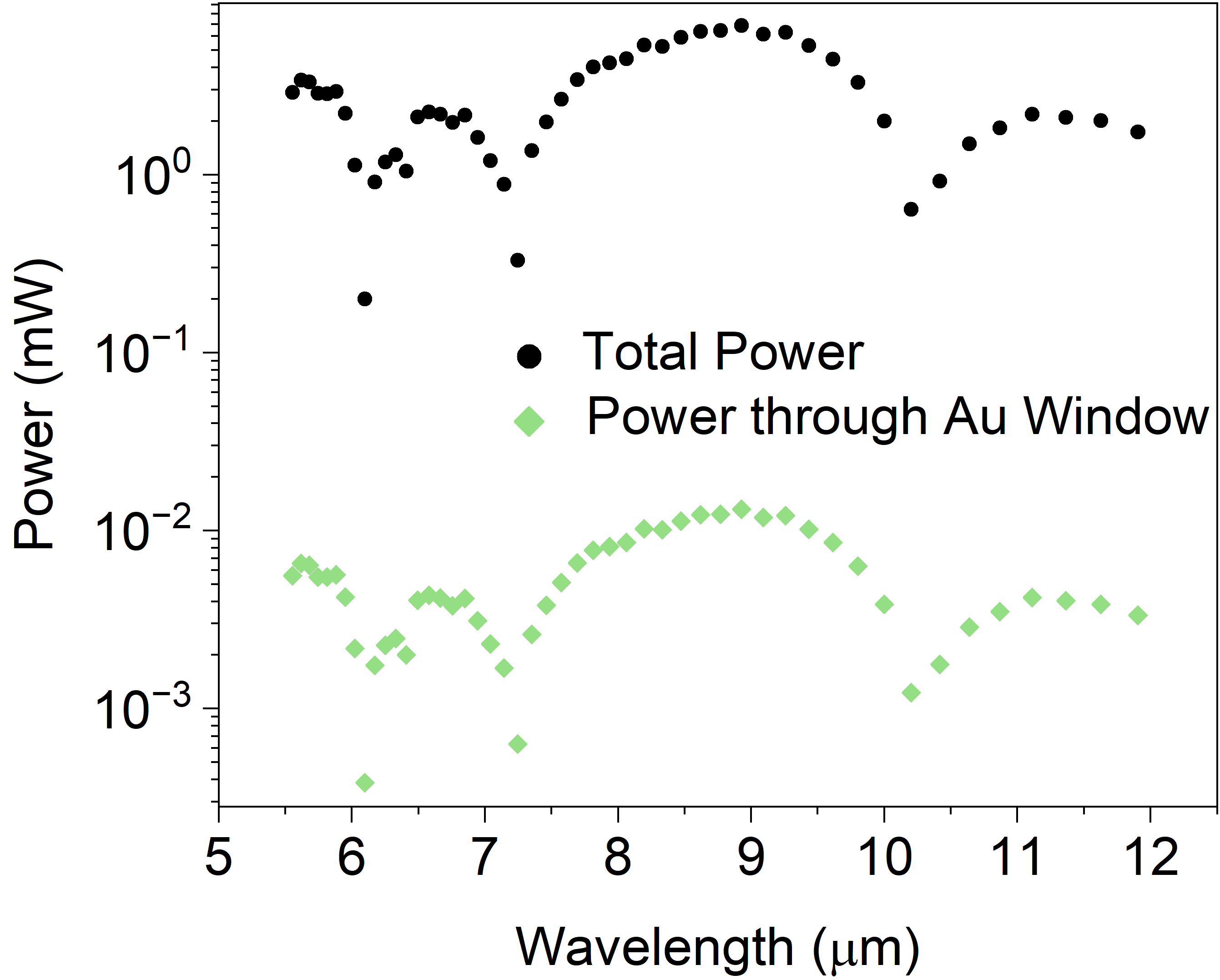
***

**Supplementary Figure 22:** Plot of the total power of the laser and the approximate power transmitted through the Au window surrounding the bolometer pixel.


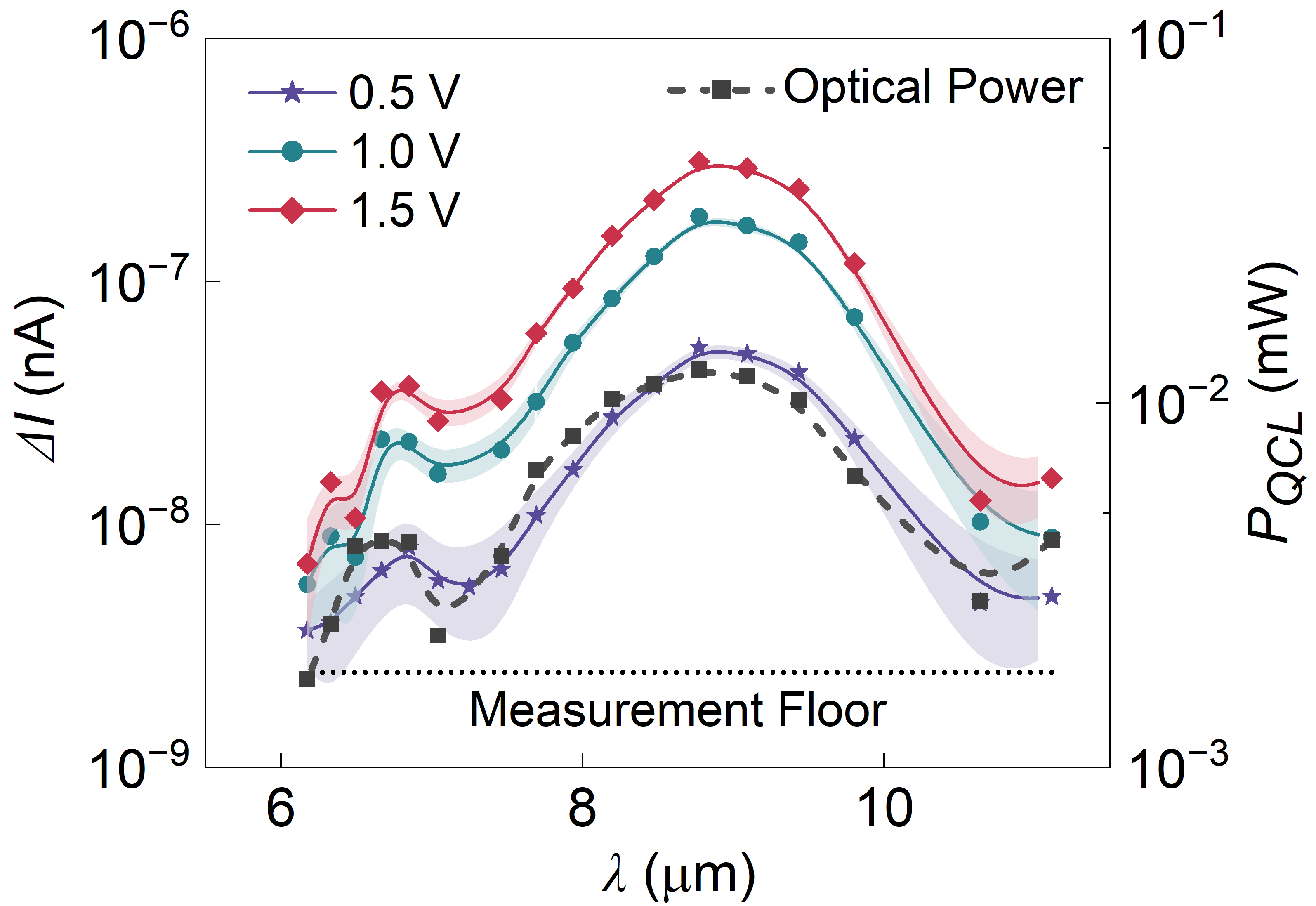


**Supplementary Figure 23:** The optoelectronic measurement data of the bolometer device with PMA having a resonance peak at 9 μm. The signal current was measured by lock-in-based detection method at room temperature and under vacuum conditions (10^−2^ mbar).


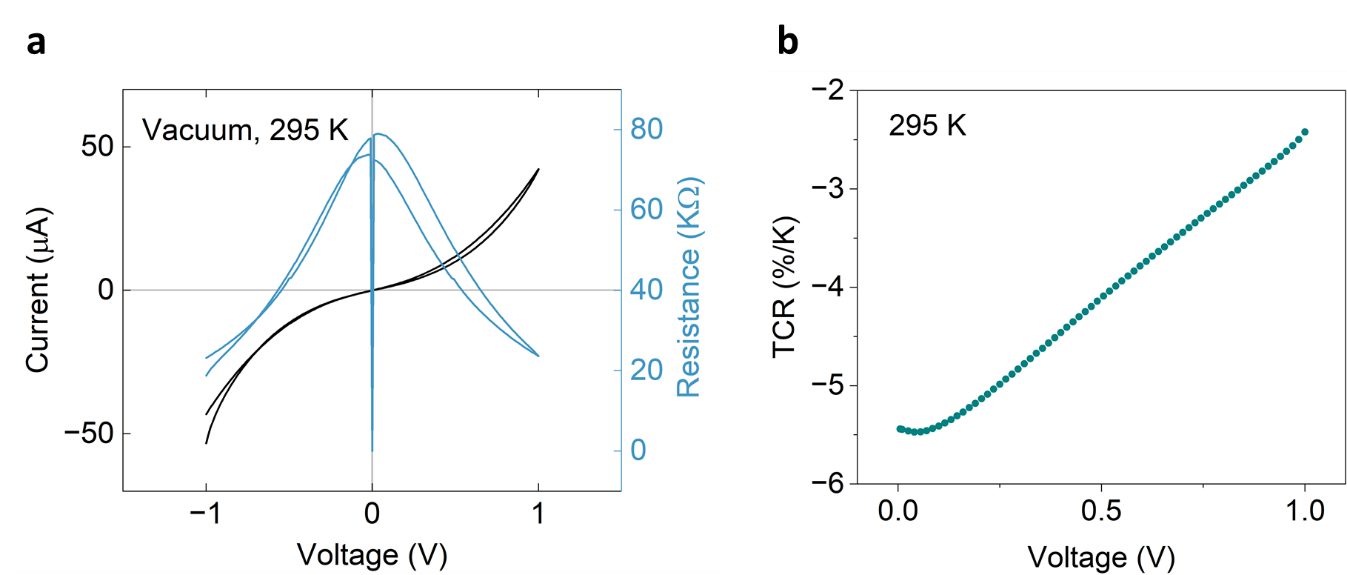


**Supplementary Fig. 24:** Thermistor characteristics of the bolometer pixel presented in the main text. a, I-V. b, TCR


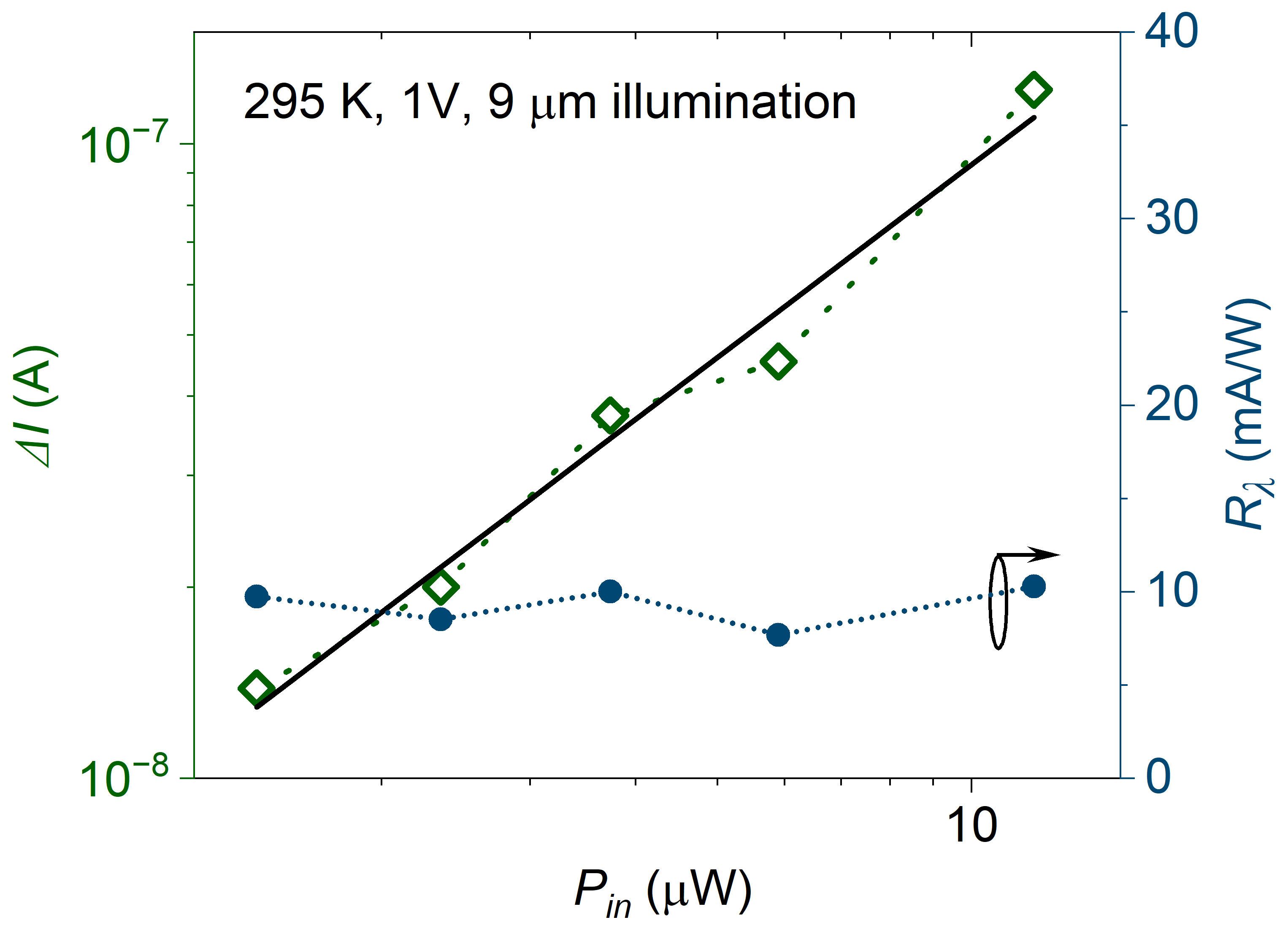


**Supplementary Figure 25:** Power dependent photocurrent and responsivity of QDPBT bolometer.


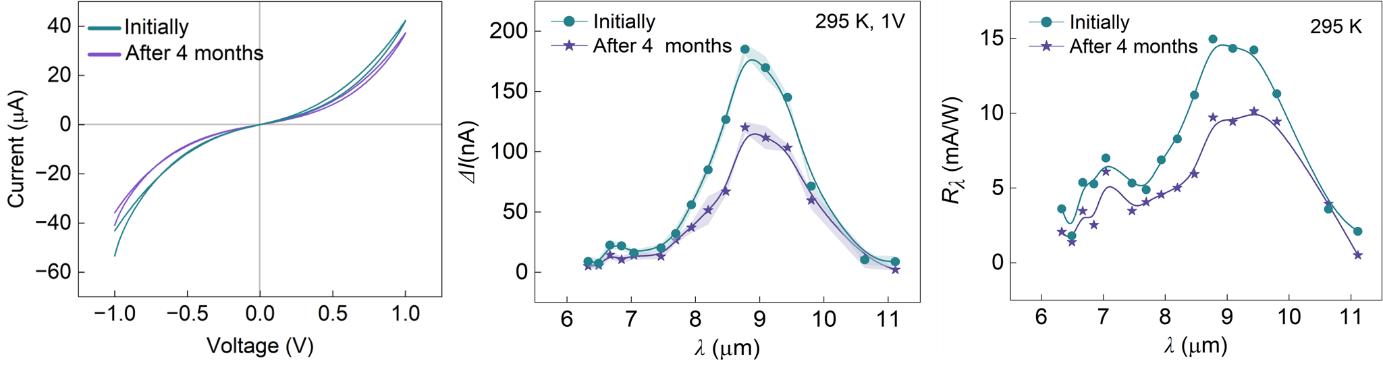


**Supplementary Figure 26:** Long-term stability of the bolometer device presented.

**
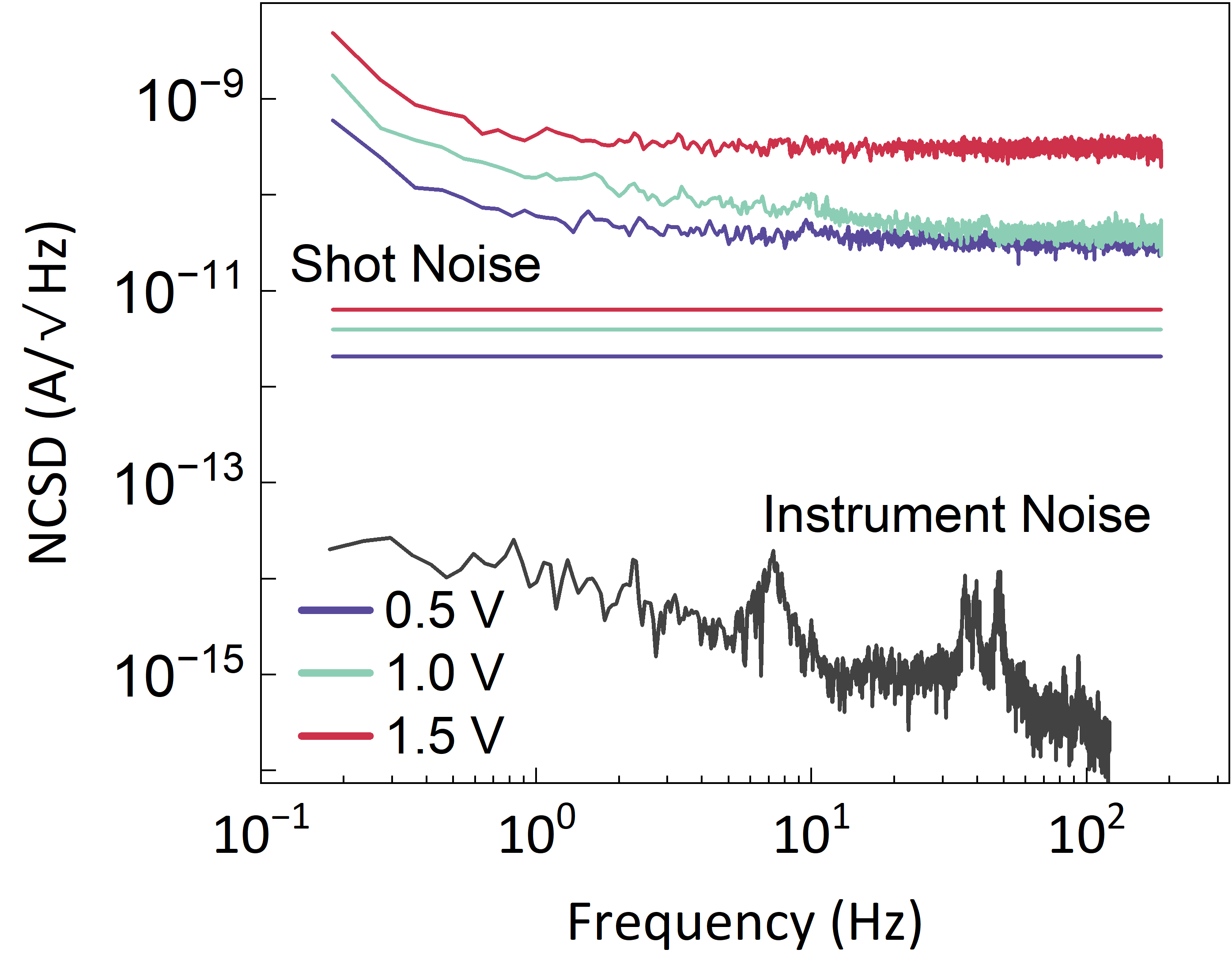
**

**Supplementary Figure 27:** NCSD measurements of the complete bolometer device after all the fabrication steps. The shot noise levels at different biases are also shown in the figure. The noise calculations were made by following the FFT method using MATLAB and using the Hanning window to ensure minimal spectral leakage due to FFT operation.

**Supplementary Note 5: Thermal Conductance (**$\boldsymbol{G}_{\boldsymbol{th}}$**) Estimation:**

The induced temperature rise $\Delta T$ due to the optical power absorption is given by:

$$\Delta T=\frac{\Delta I}{\alpha\times I_{d}}$$

where, $\alpha$ is the TCR, $\Delta I$ is the signal change upon absorption of optical power $P_{in}$, and $I_{d}$ is the device current at a given bias. For our device, at 1 V and 4 Hz, $\Delta I$ = 170 nA, $\alpha$ = ~0.025 K^−1^, $I_{d}$ = 30 µA, the induced temperature change $\Delta T$ comes out to be around 230 mK. $\Delta T$ can be further utilized to calculate the thermal conductance, $G_{th}$^[1]^,

$$\Delta T=\frac{\eta P_{in}}{G_{th}\left( 1+\omega^{2}\tau^{2} \right)^{1/2}}$$

Assuming $\eta$ = 0.8 (for our PMA), the $G_{th}$ is estimated to be around 5 × 10^−5^ W K^−1^.

1. Rogalski A, Infrared detectors: status and trends, Progress in Quantum Electronics 27 (2003) 59–210.
